# Natural Hydrogel-Based Bio-Inks for 3D Bioprinting in Tissue Engineering: A Review

**DOI:** 10.3390/gels8030179

**Published:** 2022-03-14

**Authors:** Ahmed Fatimi, Oseweuba Valentine Okoro, Daria Podstawczyk, Julia Siminska-Stanny, Amin Shavandi

**Affiliations:** 1 Department of Chemistry, Polydisciplinary Faculty, Sultan Moulay Slimane University, P.O. Box 592 Mghila, Beni-Mellal 23000, Morocco; 2 ERSIC, Polydisciplinary Faculty, Sultan Moulay Slimane University, P.O. Box 592 Mghila, Beni-Mellal 23000, Morocco; 3 3BIO-BioMatter, École Polytechnique de Bruxelles, Université Libre de Bruxelles (ULB), Avenue F.D. Roosevelt, 50-CP 165/61, 1050 Brussels, Belgium; oseweuba.okoro@ulb.be (O.V.O.); julia.siminskastanny@ulb.be (J.S.-S.); 4 Department of Process Engineering and Technology of Polymer and Carbon Materials, Faculty of Chemistry, Wroclaw University of Science and Technology, Norwida 4/6, 50-373 Wroclaw, Poland; daria.podstawczyk@pwr.edu.pl

**Keywords:** 3D bioprinting, hydrogel, biopolymers, bio-ink, rheological properties, printability

## Abstract

Three-dimensional (3D) printing is well acknowledged to constitute an important technology in tissue engineering, largely due to the increasing global demand for organ replacement and tissue regeneration. In 3D bioprinting, which is a step ahead of 3D biomaterial printing, the ink employed is impregnated with cells, without compromising ink printability. This allows for immediate scaffold cellularization and generation of complex structures. The use of cell-laden inks or bio-inks provides the opportunity for enhanced cell differentiation for organ fabrication and regeneration. Recognizing the importance of such bio-inks, the current study comprehensively explores the state of the art of the utilization of bio-inks based on natural polymers (biopolymers), such as cellulose, agarose, alginate, decellularized matrix, in 3D bioprinting. Discussions regarding progress in bioprinting, techniques and approaches employed in the bioprinting of natural polymers, and limitations and prospects concerning future trends in human-scale tissue and organ fabrication are also presented.

## 1. Introduction

Tissue engineering is an emerging discipline, aimed at regeneration, repairing or building up of functional tissues or organs similar to human organs [1]. Hydrogels are widely studied for tissue engineering applications, via the provision of matrices capable of sustaining both differentiated and non-differentiated cells, alive, in three-dimensional (3D) constructs [2,3]. A hybrid tissue or cell-engineered biological construct (e.g., half-synthetic/half-biological) is produced either to reconstruct a damaged element of the host organism or to simulate the pathophysiology of the studied tissue to reveal the molecular processes behind it [4,5].

In tissue engineering, hydrogels’ ability to crosslink in situ eliminates the need for open surgery to implant them. They can be crosslinked after the implantation process under different conditions. Both chemical and/or physical modification can be employed to induce gelation, provided that the encapsulated cells survive and proliferate afterwards [6].

The use of bio-ink containing other biomaterials may provide additional mechanical support for the bioprinted cells, helping them to organize, migrate and differentiate autonomously to form functional tissues [7]. It is, therefore, possible to manufacture physiologically complex human heterogeneous tissues in a personalized manner. Prior to implantation, 3D bioprinted patches or tissues can also be enriched with molecules of biological interest, such as growth factors, known for their regenerative properties [8].

3D bioprinting can facilitate the creation of biological structures from a bio-ink to obtain a synthetic extracellular matrix (ECM). To date, the majority of the 3D bioprinting technologies for scaffold-based fabrications can be classified under four leading categories, namely extrusion-based, droplet-based, laser-assisted and bioprinting based on vat polymerization [9,10,11]. The most common 3D bioprinter, the extrusion bioprinter, is in fact based on the same principle and involves material deposition layer by layer, typically using pneumatic, piston or screw syringes [12]. However, there are other 3D bioprinters that deposit thermal (or piezoelectric) bio-ink drop by drop (inkjet bioprinter), similar to a traditional material jet printer [13], and others using lasers as a source of energy (e.g., laser-assisted bioprinter) [14] or utilizing photo-initiators to enhance the crosslinking mechanism of polymers (e.g., vat polymerization-based bioprinter) [10,11].

Finding an appropriate bio-ink is of paramount importance in 3D bioprinting, as it provides a tissue-specific microenvironment that can support cellular growth and maturation. Among the variety of bioprinting materials employed in the manufacture of physiologically complex heterogeneous human tissues, several biomaterials have been explored, such as synthetic hydrogels (Polyethylene glycol [15,16], polyurethane [17], Poly(vinyl alcohol) [18], polylactide and derivatives [19,20,21,22]) and natural hydrogels such as collagen [23,24,25,26,27,28,29,30,31,32,33,34], fibrin [35,36,37], silk [23,32,38,39,40,41,42,43,44,45,46,47,48,49], hyaluronic acid [33,34,50,51,52,53,54,55,56,57], chitosan [24,46,58,59,60,61,62], cellulose [21,43,49,63,64,65,66], agarose [32,46,67,68,69,70], carrageenan [71,72,73], bacteria [74], etc. Additionally, some decellularized extracellular matrix (dECM) hydrogels, which are an amalgamation of various proteins in the ECM of a native tissue/organ, are considered as native hydrogels and may be used as bio-inks [29,30,75,76,77,78,79,80,81]. From a design viewpoint, a 3D bioprinted dECM scaffold has the capability to meet all clinical challenges, including some performance elements that other bio-inks do not have. This type of bioprinting material is known to have higher regenerative potential than conventional commercial hydrogels [82,83]. Crucially, the capacity of 3D bioprinting of hydrogel-based bio-inks has been demonstrated in the regeneration of several types of damaged tissues, including heart [84], cartilage [50,63,66,78], bone [85,86,87,88], muscle [81,89,90], kidney [29,89], skin [30,49,90,91,92], blood vessels [53,93,94], adipose tissue [95,96], intestinal tissue [97], liver [98], trachea graft [37], breast tissue [99], ocular tissue [100] and other engineered biological tissues [101,102].

Recognizing the potential widespread applications of hydrogels, several previous researchers have sought to undertake studies in the area [103,104]. For instance, the study of Kundu et al. [103] explored the potential of employing different celluloses as natural biopolymers for applications including wound healing. Similarly, Yang et al. [104] studied the utilization of polysaccharide hydrogels in tissue engineering. Functionalization and modification of polysaccharides enabled the formation of hydrogels, and the introduction of versatile side groups helped to regulate cell behavior. Tang et al. [105] also assessed protein-based hydrogels with respect to their common fabrication methods, properties and suitability in multiple applications, such as tissue engineering and drug delivery. A consideration of these previous studies shows that most research in this area tends to focus on only natural-based hydrogels (i.e., either polysaccharide or protein based). In this regard, the present review discusses recent progress in the design and development of hydrogel-based, natural bio-inks for 3D bioprinting in tissue engineering and regenerative medicine, in a comprehensive manner. The present study will also discuss the formulation and the use of natural hydrogel-based bio-inks and their characteristics, such as rheology, printability, etc. Additionally, the functionality of multicomponent bio-inks consisting of various protein-based hydrogels, dECM and/or polysaccharide-based hydrogels will be discussed. Finally, challenges, future outlooks and tendencies associated with the 3D bioprinting of natural hydrogel bio-inks are addressed.

## 2. 3D Bioprinting and Process Parameters

### 2.1. 3D Bioprinting

Tissue engineering and regenerative medicine have new meaning thanks to 3D bioprinting. Furthermore, 3D bioprinting has a great potential to improve the biomedical field, as it includes the design, prototyping and fabrication of 3D tissue structures that could be used for regeneration, repair or building up of functional tissues or organs similar to those of a human being. The “bioprinting material” utilized in 3D bioprinting techniques, also referred to as bio-ink, often includes living biological cells, hydrogels, chemical factors and biomolecules, to form a physical and functional 3D living structure [106]. 3D bioprinting was first demonstrated using the conventional inkjet process. The inkjet printing translates a digitalized computer image of data or character and reproduces it contactless on a specific substrate in the form of droplets [107]. In the early 1980s, a graphics plotter for precise deposition of cells and a commercially accessible inkjet printer delivered by Hewlett-Packard, employing thermal, drop-on-demand technology, were used to deposit cells using cytoscribing technology and a hydrogel solution as the bio-ink [108].

To successfully create bioprinted tissues or organ-like structures that facilitate cell proliferation, it is essential to initially generate a set of printing instructions and select suitable bioprinting materials, bio-inks (e.g., synthetic-based hydrogels, protein-based hydrogels, polysaccharide-based hydrogels and dECM-based hydrogels) and cells. The last steps, involving control of the bioprinter prior to starting the process of fabrication and quality control after printing, are also important [9].

**Figure 1:**
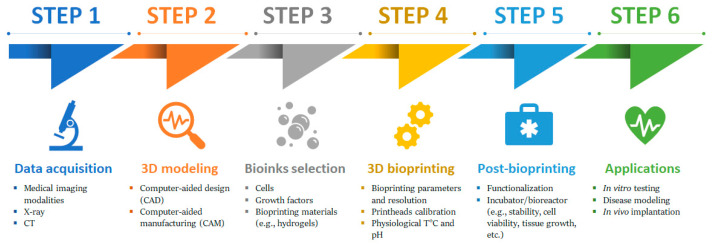
Typical manufacturing workflow for 3D bioprinting process and bioprinted tissues.

Generally, an ideal 3D bioprinting process should follow a typical manufacturing workflow for bioprinted tissues. The process of 3D bioprinting is composed of several stages, namely Pre-bioprinting (data acquisition and 3D modelling), Cell and bio-ink preparation, Bioprinting process and Post-bioprinting/applications [9,109] (Figure 1).

#### 2.1.1. Pre-Bioprinting

In this stage, a digital file for the bioprinter is created. This file contains 3D models and is obtained via the acquisition of imaging data for the 3D representation of tissue or organ. In some cases, imaging data are acquired via X-ray, computed tomography (CT), or magnetic resonance imaging (MRI) techniques or is created directly with a computer-aided design (CAD) software. The feasibility is then verified using computer-aided manufacturing (CAM) software [110,111,112]. The print file is then converted to a printer readable file, which is stereolithography (STL) [113], and the paths for the printheads are created using a process analogous to the preparation of samples for histology [114,115]. The data are then translated to enable the estimation of the material amount needed to be extruded, which depends on the desired layer height and width in accordance with bio-ink shape (e.g., droplets or filaments) [39].

#### 2.1.2. Cell and Bio-Ink Preparation

Cells from the tissue biopsy are initially isolated, expanded and differentiated in vitro. The choice of cells depends on the application and can be patient- and/or organ-specific primary or stem cells [9]. The bio-ink containing the isolated cells, growth factors and bioprinting materials is then prepared according to the physiological temperature, pH and requirements of printed structures [9]. A live-cell imaging system is used before bioprinting to ensure there are enough cells to bioprint a tissue model successfully [9].

#### 2.1.3. Bioprinting Process

Prior to the bioprinting process, an appropriate configuration of the device must be maintained and followed by setting bioprinting parameters. Nevertheless, observation during the printing process is essential to make adjustments when problems occur [116]. Depending on the structure to build, the multiple print heads are calibrated in position, and the cell-laden bio-ink is loaded into the cartridge, respecting physiological temperature and pH. When the bioprinting starts, the bioprinter follows the instructions of designed paths and deposits bio-inks, systematically building the 3D tissue or organ according to a series of 2D slices [9,109]. Bioprinting resolution is specific to the bioprinter used and the type of bio-ink used for bioprinting; usually, the greater the resolution, the longer the time of object fabrication [117].

#### 2.1.4. Post-Bioprinting

At this stage, the printed structures are usually crosslinked to enhance their stability and later examine them via microscopy imaging techniques, providing information regarding the in vivo cell distribution in a defined area or volume of the 3D bioprinted scaffold. The dispersal and the cell functionality in the construct are also checked [116]. The successfully cell-filled constructs are kept in an incubator or a bioreactor for culturing and maturation, after which the resulting artificial tissue constructs are used either for implantation or as platforms for vitro studies [118].

Having covered the underlying aspects of 3D bioprinting, the associated technologies utilized in 3D bioprinting are discussed in the subsequent section.

### 2.2. 3D Bioprinters and Technologies

Conventional additive or layered manufacturing techniques gave rise to many of the 3D bioprinting methods. However, what significantly hinders the 3D bioprinting techniques, in comparison to AM-based methods of scaffold fabrication, is attributed to the direct involvement of biological living materials during the fabrication process. Several companies are already in the business of making 3D bioprinters that are capable of printing tissues and organs of clinically relevant shape and size (Table 1).

**Figure 2:**
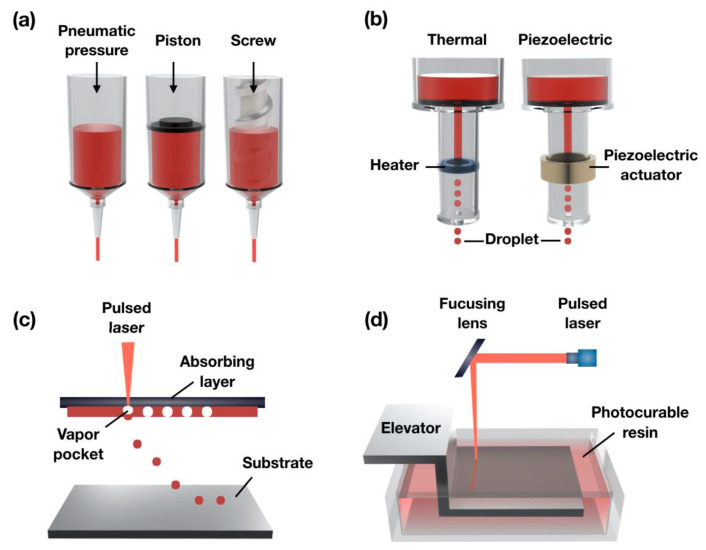
An overview of the commonly used scaffold-based 3D bioprinting: (**a**) extrusion-based, (**b**) inkjet-based, (**c**) laser-assisted and (**d**) vat polymerization-based bioprinting (Reprinted from Jeong et al., 2020 [119]. Copyright © 2022 MDPI under the terms of the Creative Commons Attribution 4.0 International License).

According to different technological approaches and bioprinting materials, the most used scaffold-based 3D bioprinting is classified as (Figure 2); extrusion or droplet-based, laser-assisted or vat-based polymerization bioprinting [9,10,11,116].

**Figure 3:**
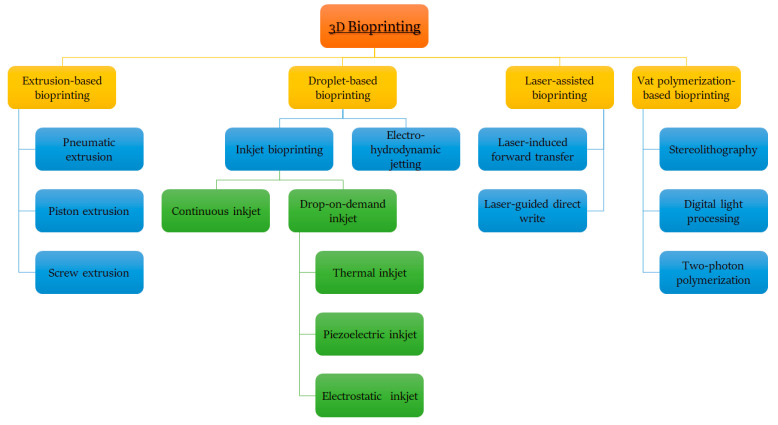
Detailed classification of the major scaffold-based 3D bioprinting: extrusion-based, droplet-based, laser-assisted and vat polymerization-based bioprinting.

Generally, bioprinters based on the extrusion process deposit bio-inks to form unbroken filaments for the assembly of 3D structures; droplet-based bioprinting creates discrete droplets of bio-inks and incrementally stacks them into 3D structures; laser-assisted bioprinting uses laser energy in the form of impulses to transfer bio-inks to a substrate in a 3D spatial arrangement; and vat polymerization-based bioprinting uses ultraviolet or infrared radiation to build 3D structures in a reservoir while using liquid photocurable bio-ink [119]. A detailed classification of the most used scaffold-based 3D bioprinting processes is shown in Figure 3 and further discussed in subsequent sections [116,120].

**Table 1:** Examples of 3D bioprinters using hydrogel-based bio-inks in tissue engineering and regenerative medicine.

Company	Bioprinter	Features	Tissues or Organs	References
ORGANOVO (San Diego, CA, USA)	NovoGen MMX™	Able to create biological tissues of the liver, kidneys, intestines, skin, pancreas and more. Includes two printheads, one for extracting cells, the other for hydrogels, scaffolds or soft biomaterials.	Kidney, tissue-engineered muscle, liver, human intestinal tissue.	[89,97,98,121]
ENVISIONTEC (Gladbeck, Germany)	3D Bioplotter^®^	Can process a variety of biomaterials (e.g., hydrogels, soft polymers, bioceramics, etc.). Used in bone regeneration, cell and organ pressure, production of cartilage and skin.	Blood vessels, adipose tissue, tracheal graft, tooth tissue, adipose tissue.	[37,93,95,96,122]
CELLINK (Gothenburg, Sweden)	Inkredible+™	Based on the extrusion principle. Equipped with dual heated printheads. Allows 3D bioprinting with different cell types and bio-inks in the same structure. Several biomaterials can be used, including those of too-high viscosity at room temperature. Equipped with a built-in UV crosslinking system.	Cartilage and skin tissue, vascularized soft tissues, skin constructs.	[49,92,123]
CELLINK (Gothenburg, Sweden)	BIO X™	Integrates three different printheads. Based on the principle of extrusion. Could design structures from any type of cell (e.g., endothelial cells, stem cells or fibroblasts). Equipped with UV-C germicide that allows sterilizing light in the printing environment.	Engineered neural tissues, skin constructs, wound dressings, bone tissue.	[57,86,92,124]
ASPECT BIOSYSTEMS (Vancouver, BC, Canada)	RX1™	Able to manufacture physiologically complex heterogeneous human tissues in a personalized way. Bioprinting of high cell densities with high viability and preserved phenotype. Uses low viscosity biomaterials.	Engineered neural tissues, brain tissue, renal tissue, 3D contractile smooth muscle tissues, neural tissues.	[125,126,127,128,129]
GESIM (Radeberg, Germany)	BioScaffolder^®^	Bioprinting of very different hard and soft biopolymers with or without cells. Design and bioprinting of porous and multi biomaterial structures for tissue engineering. Sequential bioprinting, co-axial extrusion, nanoliter pipetting.	Vaginal wall repair, periodontal tissue, cardiac tissue.	[130,131,132]
ALLEVI (Philadelphia, PA, USA)	Allevi	Uses LED photo-curing with blue and UV light. Allows working with several biomaterials (e.g., collagen, matrigel, methacrylate, graphene, etc.).	Veterinary dosage forms, bone graft, osteochondral constructs.	[133,134,135]
REGENHU (Fribourg, Switzerland)	3D Discovery^®^ Evolution	Enables fabrication in macro and nano dimensions using a single unit. Generates tissue structures analogous to those seen in nature. Provides 11 different printhead technologies with only a single instrument. Configuration and specification can be modified and adapted.	Cartilage tissue constructs engineered biological tissues.	[101,102,136]
REGENHU (Fribourg, Switzerland)	Biofactory ^®^	Adapted to a wide range of bioprinting techniques, including extrusion and droplet bioprinting techniques. - Enables work with a vast range of biomaterials, including photo-crosslinkable hydrogels, proteins and high viscosity biomaterials. Provides a system built into the laminar flow hood, which maintains a sterile environment with regulated temperature, humidity and gas composition.	Skin, air–blood tissue barrier, skin tissue regeneration, 3D tubular construct.	[137,138,139,140]
CLUSTER TECHNOLOGY (Osaka, Japan)	DeskViewer™	Based on the principle of piezo-electronic inkjet printing. Equipped with four injectors with different-sized nozzles. Able to print different kinds of cells or protein solutions. Both the volume and diameter of the drop from the nozzle can be modified and adapted.	Human tissue chips.	[141]
REGEMAT (Granada, Spain)	Bio V1	Optimized for osteochondral tissues and able to be used in other similar applications. Exchangeable printheads allow for a wide spectrum of applications.	Bone tissue, articular cartilage constructs.	[50,85,142]
POIETIS (Pessac, France	NGB-R™	Characterized by high precision and resolution. Provided with a built-in in-line monitoring system capable of controlling the accuracy of each layer applied, thus producing controlled 3D cellular structures and reproducible tissue designs.	Skin model.	[143]

#### 2.2.1. Extrusion-Based Bioprinters

Bioprinting techniques based on the extrusion process cover pneumatic, piston and screw-driven bioprinting [116,120]. Extrusion bioprinters, first introduced in 2002 [144], are the most frequently used in bioprinting, mainly due to their versatility, practicality, affordability and possibility their ability to generate large-scale 3D structures [145].

A typical extrusion bioprinter has two or more printing heads capable of extruding bio-ink composed of cells, growth factors and/or bioprinting materials (e.g., hydrogels), by applying a continuous pressure, enabling the dispersion of bio-ink filaments through a small, or even a micro-sized nozzle. The direction of layer deposition may vary between bioprinter models. In the major cases, the cartridge is fixed to a print arm moving in the z–y direction over a collector moving the *x*-axis, and this enables the creation of 3D patterns [146].

The main advantage of the relatively low speed and/or pressure extrusion lies in circumventing the harsh conditions (shear, shock, heat, etc.) that the cells may encounter in other bioprinting approaches. Other advantages of pressure extrusion include the use of a broad range of viscosities of biomaterial-based bio-inks, a high cell density and different concentrations of cells [147,148]. Disadvantages of this approach include hydrogel deformations, relatively low resolution, potential nozzle clogging and the apoptosis of embedded cells, mainly due to the induced pressure imposed within the nozzle [146].

Extrusion-based bioprinting is undoubtedly the most common modality employed in current bioprinters due to its ease of use and lower start-up and conservation costs. Extrusion-based bioprinters represent 57% of the commercial bioprinters of the global 3D bioprinting market [149,150,151].

Additionally, compatible with extrusion bioprinting are the coaxial and multi-material techniques, suitable for different sorts of applications. However, in general, the extrusion bioprinting approach has been used to fabricate 3D tissues and biological constructs including kidney [89], liver [98], blood vessels [93], tissue-engineered muscle [121], human intestinal tissue [97], adipose tissues [95,96], tracheal graft [37], tooth tissue [122], vascularized soft tissues [123], skin constructs [92], engineered neural tissues [126], brain tissue [127], renal tissue [128], cartilage tissue constructs [50,137,143], bone tissue [85] and other engineered structures [103,104].

#### 2.2.2. Droplet-Based Bioprinters

Introduced in 1988, droplet-based bioprinting approaches can be further divided into electro-hydrodynamic jetting, inkjet, acoustic or microvalve-based bioprinting [108]. Inkjet bioprinting was the first droplet-based bioprinting approach to be developed [108]. The inkjet bioprinting method can be divided into continuous bioprinting and drop-on-demand, where single droplets are deposited according to a defined path. The drop-on-demand technique is based on three different droplet generation mechanisms: piezoelectric, thermal and electrostatic [116,120].

The continuous inkjet bioprinting technique, as it requires conductive bio-inks, is not well adapted to bioprinting; moreover, the contamination risk from ink recirculation is high. On the other hand, the drop-on-demand approach is of fundamental importance in bioprinting due to the pulsed character of the printing. A cartridge is loaded with cell-laden bio-ink and then printed in well-distributed droplets, which are generated from the printhead controlled by the thermal or piezo actuator. The bio-ink droplets, if needed, are ejected through the nozzle opening by a pressure pulse inside the microfluidic chamber [152].

The main advantages of the drop-on-demand method are low costs, as the devices used are similar to the commercial equipment and can print at high speed due to the ability of printheads to work in parallel, and high cell viability [153]. Disadvantages of the drop-on-demand method include its narrow material selectivity, temperature variations during the printing process and frequent printhead clogging [152]. To alleviate existing problems and achieve better performance, hybrid cell printing techniques have been developed and studied [154].

The second most popular mode within the current bioprinters is inkjet-based bioprinting. Bioprinters of that type represent 10% of the commercial bioprinters globally. Just a few manufacturers offer inkjet printing of cells, as there is a technical challenge of obtaining uniform droplets as well as a practical challenge of obtaining higher cell densities hidden behind this technique [150].

To date, inkjet-based bioprinting has been utilized to create 3D tissues and biological constructs, including 3D replicas of cartilage [155], engineered neural tissues [126,129], brain tissue [127], renal tissue [128], 3D contractile smooth muscle tissues [125], skin tissue [137,138], air–blood tissue barrier [139], human tissue chips [141], branched vasculatures [156], liver [157] and other complex heterogeneous tissue constructs [158]. Moreover, inkjet-based bioprinting could be beneficial in areas such as wound healing, since individual droplets of cell-laden bio-ink could be used to fill empty wounds in a layer-by-layer manner, with varied cell populations applied as a function of depth [159].

#### 2.2.3. Laser-Assisted Bioprinters

Introduced in 1999, laser-assisted bioprinting shows a resemblance to direct writing methodologies [14]. Laser-assisted bioprinting includes laser-induced forward transfer, laser-induced forward transfer supported by an absorption film and direct matrix-assisted laser evaporation writing. Notably, other techniques, such as biological laser processing and laser-guided direct-write, are regarded as derived or modified versions of one of three of the first techniques [116,120].

Laser-assisted bioprinting is neither a cheap nor an easy technique. To transfer materials to a substrate, this technique employs pulsed laser energy. A typical laser-assisted bioprinter is mainly composed of a pulsed laser source, optics necessary for the beam delivery, a target in the form of a ribbon coated with the bio-ink to be bioprinted, and a receiving substrate. However, since laser-assisted bioprinting is a nozzle-free process, it is not hindered by clogging problems generated by cells or biomaterials, which characterizes some other bioprinting techniques, e.g., extrusion-based bioprinting [153].

The laser-assisted bioprinting method also has the advantage of bioprinting with biomaterials of high cell density and viscosity. It enables printing at high resolution while avoiding the high shear stress related to the material passing through a nozzle (inkjet-based bioprinting) or a needle (extrusion-based bioprinting) [160]. As an optical technique, it is possible to visually identify and position cells and biomaterials in real time for subsequent deposition. Thus, laser direct-write techniques provide an appealing alternative to bioprinting multicellular structures in space-ordered patterns with near single-cell resolution. It is a non-contact, orifice-free technique offering the ability of biological material deposition with microscale precision [161,162]. Thus, laser-assisted bioprinting can overcome the lack of precision with respect to the shape of the microscale structure, which characterizes other bioprinting techniques [163]. It also results in higher cell viability compared to inkjet and extrusion mechanisms [150].

Laser-assisted bioprinters that are not yet commercially available may be assembled depending on the desired capabilities [149]. Laser-assisted bioprinters represent 3% of commercial bioprinters in the global 3D bioprinting market, with only the POIETIS (Pessac, France) company focusing on the production of laser-assisted bioprinters [143,150] as well as built-in monitoring systems capable of controlling the quality of each layer of the bioprint, thus producing controlled and reproducible 3D cell structures and tissue models [143]. These bioprinters are suitable for fabricating complex 3D tissue constructs, including hollow tubular tissue constructs [164,165], skin tissue [166,167], bone tissue [168] and other 3D tissue grafts [169]. In addition, a patterned biomimetic human liver model using laser-assisted bioprinting was successfully developed and 3D bioprinted. It mimicked the liver lobule structure, which is difficult to fabricate using extrusion or inkjet bioprinting [163].

#### 2.2.4. Vat Polymerization-Based Bioprinters

Vat polymerization-based bioprinting was first introduced in 1984. It is an up-and-coming bioprinting technique suitable for various tissue engineering applications, thanks to its high manufacturing accuracy [170]. This bioprinting technique employs different photo-initiators during the bioprinting process to promote crosslinking, which are needed to fabricate complex, high-resolution tissue constructs [11,171].

Several vat polymerization-based biofabrication technologies, such as stereolithography, digital light processing and two-photon polymerization, have been developed to photo-shape cell embedded hydrogels into complex three-dimensional tissue constructs. These approaches involve layer-by-layer patterning of light, intended to photo-crosslink defined regions of a bio-ink consisting of a photo-crosslinkable hydrogel precursor [171,172]. The most representative one is stereolithography bioprinting, a light-based technique compatible with photo-sensitive bio-inks only [11,173]. Stereolithography was the first patented method that facilitated 3D object printing from digital data [170,174]. Compared to previous technologies, stereolithography bioprinting has several advantages. For instance, it is a nozzle-free process without the clogging problems of bio-inks. Furthermore, the printing time is independent of the complexity of the construct, since the whole pattern is projected on the printing substrate. This technique provides the highest spatial resolution of all existing bioprinting methods and is faster than nozzle-based bioprinting systems [11,173].

As vat polymerization-based bioprinting technology has found applications in the area of tissue engineering, various materials containing cells, biomaterials and photo-initiators have been developed. The possibilities of using vat polymerization-based fabrication methods for biomedical applications are numerous. In particular, vat polymerization-based bioprinting has been used to obtain cranial implants, customized heart valves, ear-shaped implants and aortas [171]. Vat polymerization-based bioprinting encapsulates cells in fabricated structures with higher cell densities [175,176,177]. It is important to note that the functionality of each bioprinting technique is also a function of the peculiar properties of the bio-ink.

### 2.3. Critical Process Parameters and Important Considerations for 3D Bioprinting Using Hydrogel-Based Bio-Inks

In general terms, bio-inks should have properties such as favorable viscoelastic and in situ gelation properties, high resolution during printing and short post-printing time for maturation [178]. Additionally, possible degradation end-products, generated during the process, must not lead to unfavorable immunological effects on cells [178]. However for the different bioprinting technologies, several unique properties of the bio-inks are required. For instance, while higher viscosities of the bio-ink may enhance the stability of the construct, highly viscous bio-inks may have unfavorable effects on extrusion pressure, with more pressure required for higher viscosities when the extrusion-based bioprinting technology is employed. Thus, bio-inks showing a viscosity of 10 mPa·s will be best suited for droplet-based printers, while extrusion-based bioprinters and laser-assisted bioprinters require bio-inks with viscosities of 30 to 6 × 10^7^ mPa·s and 1 to 8000 mPa·s [178,179], respectively. For the vat polymerization bioprinting, bio-inks with viscosities from 250 to 10,000 mPa·s are preferred. The high viscosity requirement of bio-inks employed in extrusion-based bioprinters suggests that the higher shear thinning property is necessary for such bio-inks to compensate for the higher shear stress occurring during the printing [178]. A similar requirement is necessary to ensure the proper functioning of droplet-based bioprinters [178]. Notably, the ability of a bio-ink to present thixotropy properties also suggests its suitability for utilization in extrusion-based bioprinting, since such bio-inks have the capacity to reduce their viscosity when shear stress is applied [180]. In vat polymerization-based bioprinting, bio-inks equipped with a laser-solidification mechanism are crucial [181]. Vat polymerization requires that the bio-inks also contain a non-toxic photo-initiator and display favorable viscosity and density to avoid cell decantation during the printing process [182].

The application functionality of the 3D bioprinted tissues and biological constructs is determined by printing fidelity (e.g., complexity, resolution, construct size, shape stability, etc.) and cell function retention (e.g., viability, proliferation, differentiation, tissue formation, etc.). However, these important characteristics are dependent on multiple parameters, such as nozzle diameter and geometry, the pressure applied, printing speed, volumetric flow rate, as well as the rheological properties of bio-inks [183,184,185]. Table 2 provides a simple comparison of different bioprinting techniques. Further discussions relating to the parameters that influence the performance of different printing techniques are also presented in Table 3.

#### 2.3.1. Nozzle Orifice Size, Geometry and Applied Pressure

Previous studies suggest that the applied pressure and the nozzle orifice size and geometry play a critical role in the printing outcome, since they influence cell viability in printed cell-laden hydrogels. Indeed, the percentage of cell mortality is dependent on the nozzle diameter and system pressure employed [163,194,195], with reduced cell viability observed as the printing pressure increases and nozzle aperture decreases [192].

#### 2.3.2. Printing Speed

The printing speed influences the efficiency of constructing millimeter or centimeter scale biostructures since maintaining cell viability after sustained printing duration is very demanding. Control over the printing speed may be performed electronically, and it ranges from picoliter to nanoliter per min. It is determined by the motion ability of the robot motors and is a decisive factor in both total printing time and the filament or droplet final dimensions [192].

#### 2.3.3. Volumetric Flow Rate

Volumetric flow rate, corresponding to the volume of printed bio-ink passing through the nozzle per unit of time, is essential for specifying the geometry of bio-printed filaments or droplets [186,187]. Assuming that the effects of hydrogel swelling and deformation are negligible, filament or droplet size may be estimated using a simple mathematical model developed based on the relationship between the volumetric flow rate and printing speed [186]. A high flow rate in combination with a lower printing speed maximizes the filament diameter, while a low flow rate in association with a greater printing speed reduces its size [187].

#### 2.3.4. Rheological Properties of Bio-Inks

Bio-inks’ rheological properties influence printing fidelity and cell durability. As bioprinting technology advances, rheology will become an even more important parameter for the optimization of hydrogel-based bio-inks. The major rheological properties affecting the final characteristics of the 3D bioprinted tissues and biological constructs include flow behavior, viscosity, shear stress and viscoelasticity [195].

##### Flow Behavior

**Figure 4:**
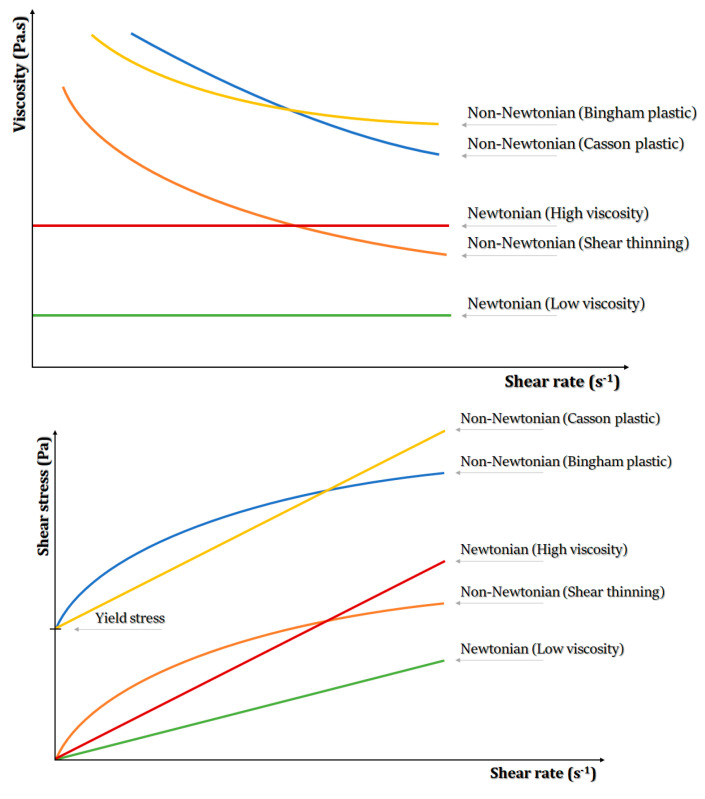
Plots of shear stress (or viscosity) versus shear rate representing flow curves of different rheological behaviors. The axes in this figure are displayed in linear scale.

The flow properties of hydrogels indicate their resistance to shear deformation and are characterized by the interplay between shear stress (or viscosity) and shear rate (Figure 4). According to this flow, behavior is generally categorized as Newtonian or non-Newtonian [201]. Bio-ink flow behavior characterization is of great significance in 3D bioprinting. Generally, hydrogel-based bio-inks exhibit non-Newtonian flow, with the preferred bio-inks reported to exhibit shear-thinning behavior to enable the bio-ink flow readily without causing clogging [202,203] while also improving the printing fidelity and stability of 3D bioprinted structures [201,204].

##### Viscosity

Viscosity constitutes an important rheological property of bio-inks, since higher viscosity may enhance the stability of the bioprinted structure at the expense of cell viability, while lower viscosity provides cells with a friendly environment but hinders printability. Furthermore, high viscosity may create clogging at the nozzle tip, so it should be adjusted based on the size of the nozzle tip. For the bio-ink formulations, viscosity can be controlled by regulating molecular weight, polymer concentration, the mass of additives, temperature and pre-crosslinking [16].

##### Shear Stress

The viscosity of bio-inks determines shear stress during bioprinting processes and thus can influence cell survival and proliferation. This is because higher shear stress levels may cause possible cell damage [196]. Thus, hydrogel-based bio-inks with low shear stress rates at moderate pressures are preferred, since they allow for ideal printing fidelity and the ability to preserve cells alive in in vitro and in vivo conditions [70,196,200,201].

##### Viscoelasticity

**Figure 5:**
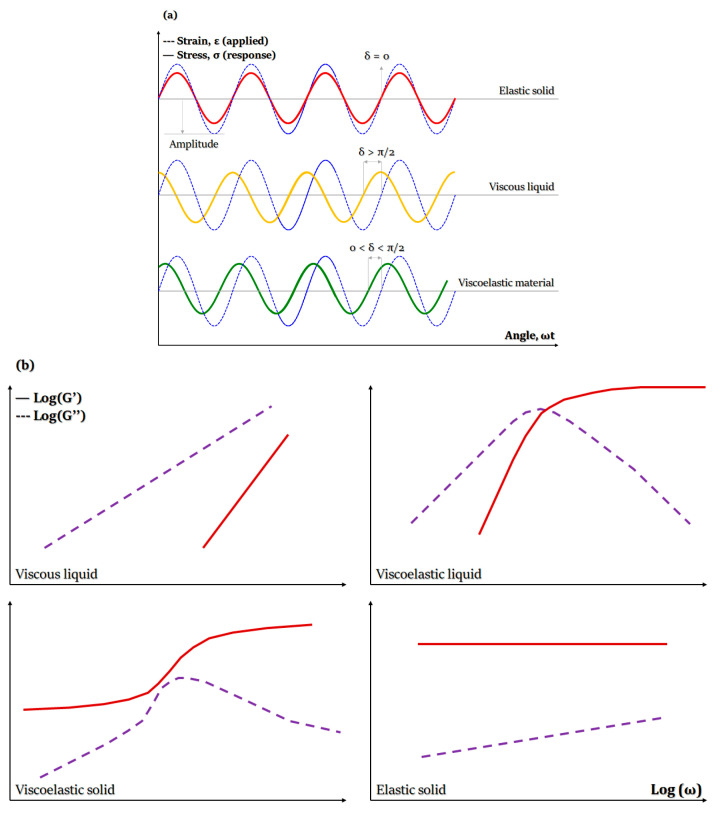
(**a**) Typical stress versus strain response for different materials during oscillatory measurements: viscous liquid, viscoelastic material and a perfectly elastic solid. (**b**) Typical behavior of the storage modulus (G’) and loss modulus (G”) as a function of frequency during dynamic mechanical testing. The values in this figure are not displayed in scale.

Viscoelasticity of hydrogel-based bio-inks is determined by undertaking dynamic measurements of storage and loss modulus as a function of shear stress, strain, frequency or time. The storage modulus, also called elastic modulus (G’), expresses the energy that is stored within the material or recoverable during each deformation cycle. On the other hand, the loss modulus, also called modulus of viscosity (G”), refers to the energy that is lost as viscous dissipation per cycle of deformation (Figure 5). Therefore, in 3D bioprinting, G’ and G” are associated with elastic shape retention and viscous flow, respectively [205,206].

Viscoelasticity can be highly dependent on the hydrogel type, concentration and applied crosslinking and plays an important role in cell-hydrogel interactions, porosity and degradation of 3D bioprinted structures. Moreover, it determines hydrogels’ structural stability and integrity and affects cell proliferation and differentiation [207]. Importantly, bio-inks with higher storage modulus exhibit more solid-like behavior, providing structural stability, but may lead to clogging and breaks in filaments. On the contrary, hydrogel-based bio-inks of higher loss moduli can be easier to work with but may lead to the formation of less stable 3D structures [206].

Another viscoelastic parameter, known as damping factor (tan(δ) = G”/G’) or loss tangent, provides valuable information concerning the relationship between viscous and elastic deformational properties. It can also help to predict the structural integrity and bioprinting uniformity during and after the bioprinting process. An ideal hydrogel-based bio-ink could develop a proper balance between the structural integrity of the hydrogel and bioprinting uniformity of the bio-ink when the damping factor ranges from 0.2 to 0.6. However, when tan(δ) is lower than 0.2 or higher than 0.6, we see nozzle blockage and bad shape retention, respectively [206,208].

## 3. Formulation and Use of Natural Hydrogel-Based Bio-Inks

### 3.1. Definition of Bio-Ink

**Figure 6:**
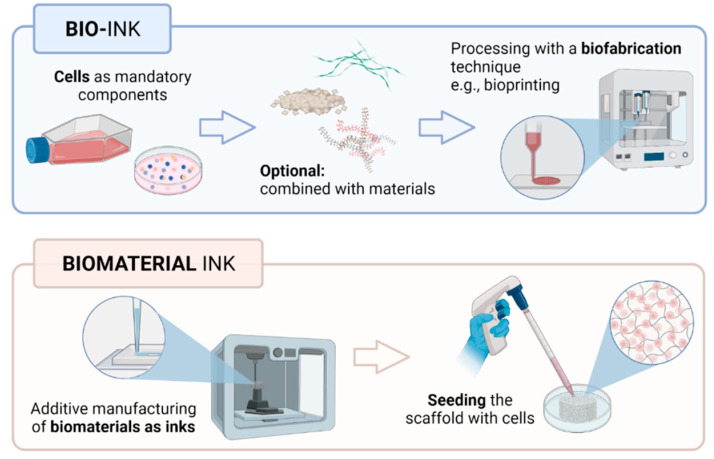
Distinction between a bio-ink (i.e., cell-laden) and a biomaterial ink (i.e., cell-free). In bio-inks (**upper** image), cells are intrinsic components of the printing formulation (e.g., seeded onto microcarriers, embedded in microgels, formulated in a physical hydrogel or formulated with hydrogel precursors). In biomaterial inks (**bottom** image), cells are introduced within the 3D bioprinted biomaterial scaffold, reducing the biological constraints on the inks (created in BioRender.com, adapted from Groll et al., 2018 [82]).

Term bio-ink refers to cells or cell aggregates that are positioned in 3D or within biomaterials [82]. In addition, in the bioprinting field, it is necessary to distinguish between bio-inks (i.e., cell-laden) and biomaterial inks (i.e., cell-free). Thus, biomaterials constituting bio-inks must act as cell carriers for the delivery of cells during formulation and bioprinting processing, while biomaterial inks can be printed but can only be seeded with cells after printing [209]. Hence, biomaterial inks do not qualify as bio-inks, as cells are usually introduced within the bioprinted biomaterial scaffold in a separate process of seeding. This however reduces the biological constraints impacting the ink properties and behavior (Figure 6).

Notably, some works have explored the use of biomaterial-free ‘inks’ composed of only cell cultures in the fabrication of 3D structures [210]. This alternative approach is in response to the risk of the included biomaterial leading to unfavorable effects on cell functions, such as cell anchorage, morphogenesis and, indeed, cell survival [211]. Such biomaterial-free inks exist mainly as 3D cultures of spheroids and organoids [212]. Spheroids are free-floating aggregates that are formed based on the presence of homotypic cell–cell adhesion. Their 3D functionality is induced either via the superficial tension-aided suspension of cultivated cells, cultivation of cells on non-adherent surfaces or cell cultivation with nanoparticles such that the structure based on cell clusters is formulated using magnetic fields [212,213]. A review of the literature also highlights several studies relating to the employment of spheroids in the 3D fabrication of constructs [214,215].

In the study of Arai et al. [214], spheroids were employed in the fabrication of scaffold-free cardiac tubular constructs. The spheroids were composed of cardiomyocytes obtained from induced pluripotent stem cells (iPSCs), endothelial cells (ECs) and fibroblasts (FBs). The tubular cardiac constructs were subsequently fabricated using a Bio-3D printer equipped with a needle array. The study was able to show that the construct responded to electrical stimuli as manifested by the variations in the beat rate. Further histological analysis established the presence of cellular reorganization in the cardiac constructs, thus highlighting the future functionality of utilizing scaffold-free Bio-3D printing techniques in the fabrication of cardiac pumps. In another study, a 3D printed culture model of HepG2 liver spheroids was developed in mini-fabricated hydrogel constructs and subsequently evaluated for drug-induced hepatotoxicity [216]. The study was able to show that 3D HepG2 spheroids provided improved resistance to nefazodone-induced mitochondrial permeability transition compared to 2D HepG2 cells. It was therefore demonstrated that the HepG2 liver spheroid platform constituted a potential tool for the appraisal of drug-induced hepatotoxicity.

Organoids are multicellular in vitro constructs that are more complex than spheroids and are designed to mimic organs. These constructs are typically composed of stem cells that are either pluripotent or are adult stem cells recovered from specific organs (i.e., liver, stomach, etc.) [212,213]. These organoids exploit the self-organization ability of stem cells to facilitate the creation of multi-cellular tissue proxies [217]. For instance, in the study undertaken in [218], a 3D heart-like organoid struct was fabricated via the encapsulation of free-suspended human pluripotent stem cells in Matrigel, after which biphasic WNT pathway modulation with small molecules was employed to initiate cardiac differentiation. The cell differentiation to produce cardiomyocytes enabled the development of a 3D heart-like structure characterized by cell layer patterns and an endoderm structure. The 3D heart-like organoid was shown to be able to replicate the heart tissue with respect to its early heart development morphology, epicardial layer and endothelial cell network. Crucially, since these constructs of organoids and spheroids do not require the introduction of biomaterials, further discussions of these so-called “biomaterial-free inks” are outside the scope of the present study, and they will not be discussed further, since biomaterial-based bio-inks constitute the focus of the present study.

Most bioprinting studies use biomaterials that are hydrogel precursors for the formulation of bio-inks as they might be crosslinked into hydrogels in the post-fabrication gelation process. Examples include polyurethane synthetic-based hydrogels [17], gelatin protein-based hydrogels [28] and alginate polysaccharide-based hydrogels [45,219]. In addition to a recent intermediate approach in the bioprinting field, pre-crosslinking of hydrogel precursors’ solution to a higher viscosity state is often applied. Later, after the fabrication process, it is followed by a complete crosslinking to fully stabilize a printed structure [16].

Hydrogel materials are the most commonly used for obtaining bio-inks; however, in general, bio-inks are not narrowed down to molecular solutions of hydrogel precursors [220]. Bio-inks can also contain microcarriers [221], nanoparticles serving as drug-releasing platforms [222] or nanofibers improving rheological and mechanical characteristics [48]. In addition, microgels that are loaded with cells [223] or microspheres [126] can be used as bio-ink components, endowing it with additional functionalities.

### 3.2. Hydrogels and Tissue Engineering

Tissue engineering techniques involve the cultivation of living cells on a 3D structure and are characterized by three requirements: (1) the availability of a matrix suitable for transplantation and maintenance of cells; (2) supporting the repair of cells that form a functional matrix; (3) availability of active biological molecules, e.g., cytokines and growth factors, facilitating the formation of new tissues.

The synthetic matrix is the structure on which the tissue architecture must organize. The cells are either those of the host, which will proliferate on the synthetic matrix in vivo, or the cells of the host cultivated in vitro on the matrix and then secondarily autografted to the patient. Cells are responsible for regenerating new tissue by synthesizing new ECM. The synthetic structure acts as a framework, providing mechanical stability and guidance for 3D cell growth. The cell/hydrogel interaction is therefore a crucial factor for the success of an application in tissue engineering.

**Figure 7:**
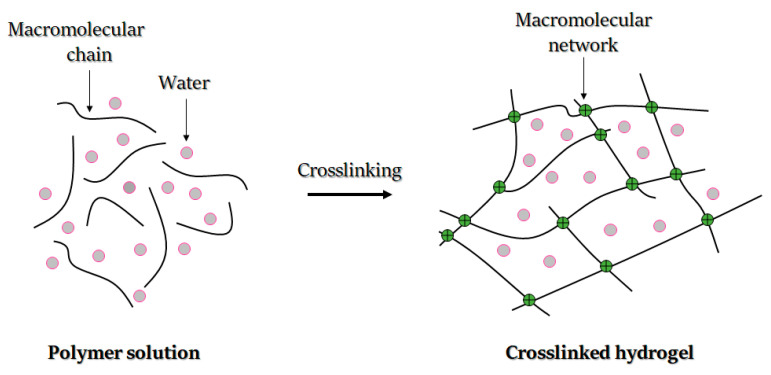
Principle of the hydrogel formation.

Hydrogels are defined as hydrophilic and crosslinked polymers that can absorb and swell in water and biofluids and transform into insoluble 3D networks. Hydrogels can be obtained from a variety of water-soluble materials, including both synthetic and natural polymers, proteins and other molecules. Their structure is mainly determined by a crosslinking process, during which an insoluble network in environmental biofluid is formed. Furthermore, the network stays in balance in an aqueous environment due to the balance between elastic forces of the crosslinked polymer and osmotic forces coming from the liquid (Figure 7).

The chemical structure composition and crosslinking density define the structure’s swelling rate and permeability. Hydrogel crosslinking endows it with an elastic response when subjected to stress. Furthermore, the structure’s elasticity and the presence of a high amount of water enables resemblance to diverse biological tissues, which therefore can be used for a wide range of biomedical applications [224].

The first hydrogel was synthesized to produce an ideal and biocompatible product [225]. The obtained hydrogel was used for the engineering of a soft contact lens. Specifically, in order to obtain a hydrogel, the molecular chains of 2-hydroxyethyl methacrylate are linked together by consecutive chemical bonds to form a uniform molecular microstructure in compliance with the following four Wichterle design criteria: (i) to avoid solubilization of hydrogel macromolecules in biofluids; (ii) to form a stable chemical and biochemical structure; (iii) to achieve high permeability of nutrients and biological residues; and (iv) to reconstitute physical features analogous to native biological tissues [226].

Based on the four Wichterle criteria [226], synthetic matrices must be compatible with biological materials and must adjust their shapes and structures to the target tissue. Hydrogels must also maintain close proximity to tissues with minimal adhesive effect. Additionally, synthetic matrices must be able to envelop cells and promote cell proliferation without damaging them under the effect of osmotic pressure. Synthetic matrices must be therefore highly porous to promote the diffusion of nutrients and metabolites among cells and the surrounding environment [227].

In tissue engineering, some hydrogels can crosslink in situ, which makes it possible to perform minimally invasive operations and avoid open surgery [21]. Hydrogels can be also crosslinked under different conditions, provided that the embedded cells survive the chemical or physical transition associated with the gelation [59].

A selection of characteristics, currently studied in the scientific literature, are discussed in the following sections, with attention to crosslinkability, biocompatibility, cell viability, swelling, diffusion, degradability, printability and mechanical strength.

#### 3.2.1. Bio-Ink Crosslinking Ability

Crosslinkability refers to how easily a material can be crosslinked and constitutes a fundamental factor in hydrogel formulation, shape and degradation. For successful biomedical applications, the control of crosslinking is crucial. In tissue engineering, crosslinking can be divided into chemical or physical types [228], which aids in enhancing the printability of bio-inks. An example of chemical crosslinking is covalent crosslinking, which can be initiated by radical polymerization, enzymatic catalysis, high energy irradiations (gamma radiation) or condensation reactions [228]. When chemical crosslinking should be achievable via radical polymerization, polymers generally require modification by adding polymerizable units. For instance, acrylate has been added to functional groups of polyethylene glycol to facilitate covalent associations in the polymer. Radical polymerization may be initiated by light systems [229]. In such light systems, photo-polymerization is achieved in the presence of a photo-initiator [228], with such light-curing considered ideal for clinical implantation, since it strengthens the three-dimensional and temporal–spatial control of the hydrogel. The inks can thus be injected, formed and solidified in situ [230]. This concept is compliant with non-invasive surgical procedures and can be used in craniofacial surgery with arthroscopic light-curing using a fiber optic light source [231].

Physical crosslinking, on the other hand, avoids the use of potentially harmful chemical crosslinking agents and may be used in the creation of biomimetic hydrogels using bioactive factors. The resulting hydrogels have been reported to exhibit unquestionable compatibility with both cells and fragile molecules [228]. Moreover, the presence of hydrogen bonds, hydrophobic interactions and Van der Waals forces facilitate physical crosslinking [232].

Physical crosslinking is also demonstrated in molecular self-assembly, which refers to a molecular construction following a sequence of activities to form a stable and well-defined network; the reverse crosslinking is prevented by physical interaction between polymer chains [232]. In molecular self-assembly, gelation kinetics is affected by the length and number of coiled-coil strains. Electrostatic and hydrophobic interactions maintain self-assembly properties and thermal stability. However, they may be tuned by manipulating the length of amino acid sequences and coiled-coil domains [233]. In addition, hydrophobic interactions can induce thermo-sensitive gelation due to temperature changes [228]. Another type of physical crosslinking occurs through ionic interactions. In this case, the polymer solution generally forms a hydrogel through the creation of ionic bonds in the presence of divalent or polyvalent cations. The main feature of ionic-sensitive inks is their ability to form reversible gels of great water-absorption capacity [234]. The ionic interactions are weaker than covalent crosslinking, and the hydrogels formed undergo rapid solubilization in physiological solutions [234].

#### 3.2.2. Bio-Ink Biocompatibility

The biocompatibility is the ability of the biomaterial to perform a specific role with a suitable host response [235]. For a more global assessment of the hydrogel-based bio-ink biocompatibility, we must consider the polymer, additives, residues and/or manufacturing contaminants and degradation products, as well as the interaction of all the components and characteristics of the finished product.

In addition, there is no absolute definition of biocompatibility, since the world of biomaterials is constantly evolving. However, from a theoretical standpoint, the desired host response is any positive interaction between the material implanted and the tissue remaining in close contact with it. Biocompatibility is a bidirectional, dynamic process involving the temporal reaction of host to material and material to host [236].

#### 3.2.3. Bio-Ink Cell Viability and Proliferation

Hydrogels fulfil a number of key roles in in vitro tissue engineering and are used to develop a controlled extracellular environment to study 3D cell/cell and cell/ECM types of interactions. The design and synthesis of new tissues with specific properties demand extensive knowledge of how cells interact with other cell types and how they may respond to other bioactive agents and the microenvironment.

The presence of the bioactive factors (e.g., bone morphogenetic protein, growth and differentiation factors, transforming growth factor, etc.) within an ink can enhance cell viability and proliferation. It has been observed that after incorporation of bioactive factors into bio-inks where cells are cultured, cell proliferation, production of extracellular matrix and collagen were increased compared to hydrogels without bioactive factors [237,238].

Cells’ ability to proliferate in inks depends on the type of ink, its concentration and the time after encapsulation. Another factor affecting cell viability is the bioprinting method, as the mechanical disturbances caused by the bioprinting process are also reported to affect cell viability [192].

In addition, the bioprinted dECM retains one of the greatest cell viability levels among bio-inks; over 95% cell viability has been noted [83]. Cell aggregate-based bio-ink materials, if small, can sustain high cell viability. However, cell viability rates in the core decrease radically when the aggregate size increases [145].

#### 3.2.4. Bio-Ink Printability

Printability of bio-ink is related to both the bio-ink formulation and its interaction with the substrate during printing, which, if well adjusted, results in printing an accurate, high-quality 3D pattern [9]. Printability is usually related to the surface tension of the supporting structures and affects attachment proliferation and differentiation of cells. In 3D scaffold fabrication, the matrix ink should maintain surface tension in the vertical direction and also have a large contact angle. Furthermore, for an ink with a high hydrophilic character, a substrate should exhibit a hydrophobic nature [239].

Bio-ink printability is measured based on its processability and the mechanical properties of the 3D construction after bioprinting [197]. An important aspect in evaluating the printability is the rheological measurements. Generally, when a bio-ink exhibits pseudoplastic, shear-thinning behavior, it may be classified as a printable material. Moreover, printability is affected by the crosslinking mechanism, the surface tension, thermal conductivity and other rheological properties such as viscosity and yield stress [206].

#### 3.2.5. Hydrogel Water Content and Swelling Behavior

An important feature of hydrogels applicable for tissue engineering is their ability to absorb body fluids. Moreover, they should be capable of permeating and transporting nutrients and metabolic products. The swelling properties of the hydrogels are among the most crucial parameters of tissue engineering that define the physical properties of hydrogels. The swelling is inversely proportional to the crosslink density and depends strongly on the chemical structure of the hydrogel [240].

In 1943, Flory and Rehner were the first to correlate crosslink density with polymer swelling in an organic solvent to quantify rubber characteristics [241]. In this model, swelling is considered to be a balance between the intrinsic elastic forces in the polymer structure and the thermodynamic forces due to the interaction between the polymer and the solvent. In 1977, the Flory–Rehner theory was modified by Peppas and Merrill who proposed another model applicable to hydrogel synthesis from polymer solutions [242]. The variation of chemical potential in the system with water is mostly due to elastic forces [227]. Moreover, the chemical structure impacts the swelling, as favorable chemical groups are present within the hydrogel. Generally, hydrogels with a majority of hydrophilic groups swell faster than those with hydrophobic groups [227].

In terms of smart hydrogels whose volume changes depend on the surrounding conditions, swelling can be affected by several parameters, like pH, temperature or the crosslinking mechanism. A swelling equilibrium can be determined experimentally or calculated theoretically. By accurately measuring equilibrium swelling, it is possible to determine crosslink density, network mesh size and diffusion coefficients. Empirical methods for measuring hydrogel swelling rate include gravimetric measurements after immersion in liquid and measurement of dimensional changes. Swelling measurements are the established standard, but in certain applications, as a sustained release of active substances, dynamic measurements can be more suitable [243].

#### 3.2.6. Hydrogel Diffusion and Solute Transportation

Controlled diffusion and solute transportation are among the key challenges in developing 3D scaffolds for tissue engineering. The ideal solution to facilitate tissue growth is to place a scaffold in the structure and pump with a culture medium rich in nutrients to deliver to the cells at the same time as removing waste products [244].

The rate of diffusion of the solute is an important parameter for determining the kinetics of the release of active ingredients, the transport of nutrients and wastes in tissue engineering. The diffusion of nutrients, gases, wastes or other solutes depends on a multitude of factors, including the morphology of the macromolecular network, the water content, the composition of the hydrogel type and its concentration, the degradation kinetics and the rate of swelling. These fundamental factors can be combined to create chemical effects or friction effects that slow the diffusion of the solute. A chemical effect describes the force of attraction between the solute and the hydrogel matrix, while the physical size of exclusion represents the primary frictional effect on diffusion through a hydrogel [245].

#### 3.2.7. Hydrogel Degradability

The degradability of hydrogels is related to hydrogel type, concentration, employed crosslinking processes, temperature, physiological conditions (in vitro and/or in vivo) and the presence of additional constituents. Undoubtedly, the degradation rate of the 3D cell-laden hydrogels should be matched to the desired biomedical application [239]. This remains a major challenge, as it is difficult to match the appropriate functional and mechanical properties of a hydrogel to a specific tissue characteristic. Moreover, cellular components should be able to replace the hydrogel, within the time of the degradation process, with newly formed ECM constituents, therefore facilitating tissue remodeling. Thus, the degradability of the hydrogel should be carefully tuned after taking into account the characteristics of the target tissue [239].

#### 3.2.8. Hydrogel Mechanical Properties

The mechanical properties that characterize hydrogels are crucial, since hydrogel-based scaffolds are expected to provide a stable condition for cell attachment, proliferation and differentiation and thus promote ECM production [246]. Of these mechanical properties, the most important are strain, shear stress and elastic modulus. It is well known that dynamic interactions between cells and hydrogels can significantly influence cell adhesion to the hydrogel matrix [247]. Under the influence of stress, the swollen hydrogel should show elastic behavior [227]. The mechanical properties of a hydrogel-based scaffold are defined by scaffold geometry, ink inherent properties and type of bulk polymer. These properties may also change with hydrogel concentration.

For example, polymer chains exhibiting higher crystallinity typically demonstrate higher tensile strength. However, if the processing method reduces the crystallinity of the polymer, the strength of the hydrogel is not free of its impact, and hence the lifespan of the hydrogel is also compromised.

### 3.3. Natural Hydrogel-Based Bio-Inks

#### 3.3.1. Protein-Based Bio-Inks

##### Collagen

Collagen is hailed as one of the most often-used biopolymers in biomedical research and cell cultures. It is undeniably the most essential component of most types of tissues’ ECM. Collagen is a cationic flexible polymer and is considered as the main structural protein in vertebrates, which primarily contain hydrophobic peptide motifs. The isolation and purification of collagen is well established, particularly for collagen type I [248,249].

Collagen allows the formation of robust and biodegradable 3D hydrogels as a result of its triple-helix structure and low antigenicity, excellent biocompatibility, low immunoreactions, clear association with other biological species and polyelectrolyte behavior [250,251,252]. Collagen-based hydrogel scaffolds have been proven useful in many biomedical applications, e.g., corneal substitutes [251,252], wound healing [249], bone tissue engineering [248] and the 3D bioprinting of cellularized structures [19,20,21,22].

According to Osidak et al., due to the biocompatibility of collagen, it is believed to be a promising material for 3D bioprinting [20]. The printability of collagen-based bio-inks has also been shown to be, irrespective of the cell density, absent of side effects in terms of the functionality or viability of printed cells [19]. A further review of the literature shows that type I collagen, which is the fibril-forming subfamily of collagens, characterized by three alpha-helices, is widely employed in 3D bioprinting [253,254]. In spite of the favorable biocompatibility of collagen, its employment in direct 3D bioprinting is limited by poor mechanical stability, especially when combined with cells or tissue spheroids, and slow gelation rate at physiological temperatures [20]. These limitations hinder its capability to maintain structural integrity once extruded. To resolve these issues, two major strategies have been employed in the literature, namely the use of sacrificial supports, which are removed after printing, and the modification of bio-ink characteristics using concentration or crosslinking strategies [253]. The use of such sacrificial supports was highlighted in the research undertaken by Moncal et al. They proposed a bio-ink composed of type I collagen and Pluronic^®^ F-127, with Pluronic serving as a sacrificial material in bioprinting operation due to its thermoreversibility and extrudability [255]. The study highlighted the viability of utilizing Pluronic as the sacrificial support, since it could readily diffuse out of the constructs without disrupting collagen fibers [255]. Another study by Stratesteffen et al. showed that blending methacrylated gelatin with collagen facilitated the creation of bio-inks that were equipped with drop-on-demand 3D printability for constructs characterized with favorable biological and rheological properties, while also promoting angiogenesis [256]. The improvement in the properties of collagen via crosslinking was demonstrated in another study [22]. Kim et al. investigated the use of genipin as a crosslinking agent with collagen-bio-ink at the optimal processing condition of ∼1 mM and 1 h of incubation in genipin solution [22]. The group was able to show that the modified collagen bio-inks could be employed in fabricating three-dimensional, pore-linked, cell-laden constructs comprising osteoblastic cells and human adipose tissue stem cells. In another study, riboflavin-induced photo-crosslinking of collagen was demonstrated [257]. The study was able to show that the riboflavin-induced photo-crosslinked collagen was characterized by improved mechanical properties and displayed a favorable delay in the enzyme-triggered collagen scaffold degradation. Apart from the introduction of crosslinking agents, collagen properties for bio-ink application can also be improved via the imposition of temperature changes [178]. The approach of utilizing temperature changes for the improvement of the properties of collagen-based bio-ink was demonstrated by Ahn et al. [258]. In the study, printing using collagen-based hydrogel was achieved with a direct cryogenic plotting method, for the deposition of low-viscosity hydrogel. The hydrogel was used in the fabrication of a hierarchical 3D scaffold with a controllable size of pores. According to the study results, the obtained scaffold showed elevated initial cell attachment and compactness between scaffold pores. In spite of the strides in research into collagen-based bio-inks, Marques et al., among many others, have stated that more investigation is required to further improve the applicability of collagen-based bio-inks [259].

##### Gelatin

A water-soluble protein, gelatin, is produced by partial hydrolysis of collagen, extracted from the boiled bones, skin and connective tissues of animals such as domesticated cattle and pigs, and therefore may differ in terms of the molecular weight (20 kDa < Mw < 250 kDa). Gelatins may be type A or type B, depending on whether it is produced via acidic or basic hydrolysis, respectively. These treatments cause de-amidation of asparagine and glutamine residues, increasing the number of aspartic and glutamic acids, respectively [260].

Gelatin is a peptide sequence mixture, soluble in warm aqueous solutions while preserving the ability to form simple gels via hydrophobic crosslinking at low temperatures. Unfortunately, the melting point of gelatin gels is in the range of 30–35 °C, thus limiting its use in applications undertaken at physiological temperatures or higher. Because of this limitation, gelatin frequently requires secondary chemical modification, alternative crosslinking processes or integration with different polymers or proteins prior to the implementation in 3D cultures [23,24,34,96,124]. Gelatin may be loaded with biomolecules, since its intrinsic features enable the control of drug loading and release kinetics via the modification of the crosslinking and the gelatin molecular weight [261]. Moreover, a variety of biomedical applications such as cell encapsulation mention the use of gelatin-based hydrogels for cell encapsulation [262]. Gelatin for wound healing approaches may be loaded with biomolecules due to its intrinsic features, and it can offer the possibility of controlling both drug loading and release kinetics with control of the crosslinking and the gelatin molecular weight [261]. Gelatin-based hydrogels may be employed for nerve regeneration [263], soft tissue reconstruction [264], bone repair [265] and 3D bioprinting of cellularized structures.

Although gelatin is recognized as a good candidate for bio-inks due to its biocompatibility and biodegradability, its use is limited by low-printability concerns. Several studies have therefore employed gelatin only after the incorporation of different crosslinking agents [25]. The improvement of gelatin’s rheological properties may also be achieved via blending with other components, as demonstrated in a previous study by Shin and Kang [266]. They prepared mixtures of gelatin containing hyaluronan and glycerol as additives, which were evaluated for their printability. The study was able to show that the mixture containing 10 mg and 20 mL of 300/90–100 bloom gelatins, 3 mg/mL of hyaluronic acid and 10% *v*/*v* glycerol leads to a uniform bio-ink with excellent printing resolution. The gelatin-based bio-ink was shown to be capable of fabricating a line of approximately 200 μm in width, which retained cells while accurately localizing in the 3D structure. Gelatin rheological properties may also be improved using crosslinking agents such as tyrosinase and genipin, which facilitate enzymatic and chemical crosslinking approaches, respectively [267,268,269]. The enzymatic crosslinking using tyrosinase was observed to lead to significant increments in the molecular weights, enhanced in the presence of phenolic molecules, and facilitated enhanced stability of a crosslinked network of gelatin [270,271,272]. Gelatin modification by metacrylation has also been extensively employed in the fabrication of extracellular matrix-derived biopolymers, able to be chemically crosslinked via radical-induced reactions [273,274]. Crucially, while the enzymatic (i.e., tyrosinase) and the chemical gelation mechanisms have been shown to increase the stability of gelatin, high cost and cytotoxicity concerns have so far limited the acceptability of enzymatic crosslinking and chemical crosslinking to enhance gelatin properties. Photo-crosslinking was therefore suggested as more appropriate when gelatin is to be employed as bio-ink in cell printing [130]. In line with this suggestion Duchi et al. investigated the photo-crosslinking of Gelatin-methacryloyl/hyaluronic acid methacryloyl (GelMa/HAMa) and discovered that bio-ink facilitated the generation of core-shell structures of GelMa/HAMa scaffolds [275]. These gelatin-based bio-scaffolds were shown to present stiffness of nearly 200 kPa after 10 s of exposure to a UV-A source (to 365 nm, 700 mW/cm^2^). Interestingly the bio-scaffolds were also able to retain high cell proliferative capacity, with over 90% of viable stem cells maintained. Similarly, in another study, a novel hybrid system was developed and consisted of gelatin macromers synthetically modified with methacrylate [276]. The novel hybrid system facilitated the photo-encapsulation of cells while maintaining mechanical integrity.

##### Fibrin

Fibrin, as the name indicates, is a fibrous protein participating in the clotting of blood. It is comprised of fibrinogen monomers that are polymerized spontaneously in the presence of thrombin and further crosslinked by the transglutaminase activity of the blood coagulation factor XIII-A [277].

In the human body, fibrin biopolymer plays a pivotal role in wound healing cascade and also tumor growth. Due to its fast crosslinking rates, fibrin gels in glue-like form have been extensively used in the clinic as a hemostatic agent, sealant and surgical glue [278].

The hydrogels based on fibrin, which is formed by the polymerization of fibrinogen, were employed in tissue culture for various cell and tissues types. Later, they were used in the tissue engineering of scaffolds for regenerative medicine applications [279]. Additionally, these fibrin-based hydrogel structures have also been applied to promote bone growth and healing [280] and neuritis extension [281]. The literature highlights that fibrin (fibrinogen) is a biomaterial that is characterized by good biocompatibility, biodegradability, and tunable mechanical and nanofibrous structural properties [253]. In addition to these favorable characteristics of fibrin, it is also regarded as a preferred choice for bio-inks, because its non-linear elasticity facilitates communication between cells [253]. In this regard, a bio-ink based on fibrin was employed in the fabrication of complex and functional cardiac tissue constructs, which were able to contract synchronously and respond to adrenaline and carbachol stimulation [282]. The use of fibrin also provided guidance to Schwann cells’, facilitating cell alignment, growth and neural tissue formation [282]. Cubo et al. also employed plasma-derived fibrin, and together with fibroblasts and keratinocytes, used it for the bioprinting of skin substitute, which was determined to recapitulate native skin when tested in vivo [283]. The use of fibrin for 3D biofabrication and bioprinting may, however, be limited by its poor mechanical properties [284]. For instance, enzymatic treatment of fibrinogen, using thrombin, to produce fibrin hydrogel was characterized by high biocompatibility and degradation ability, but presented poor mechanical properties [285]. Additionally, the high viscosity of fibrin in pre-polymer form may hinder proper ink extrusion and the ability to maintain shape fidelity [284]. To facilitate the efficient use of fibrin-based bio-inks, several approaches have been explored in the literature. For instance, gelatin may be combined with fibrin to enhance rheological properties when used in the fabrication of 3D structures [286]. Xu et al. demonstrated that a gelatin/fibrin mixture in the mass ratio of 1:1 presented excellent elasticity modulus and compressive strength and could be used in the fabrication of complex cell/matrix constructs using automated rapid prototyping techniques. The gelatin served to improve the rheological properties of the fibrin-based material due to its gelation capability at room temperature and its capacity to behave as a fluid at high shear and as a gel at low shear [284]. Additional biomaterials may also be introduced to further enhance mechanical stability. For instance, another study by Xu et al. employed the biomaterial mixture of gelatin/alginate/fibrinogen to assemble adipose-derived stromal (ADS) cells and complex in vitro 3D models, fabricated with gelatin/alginate/fibrin hydrogel [287]. Additionally, the research of Rutz et al. covered the development of multimaterial bio-ink from polyethylene glycol and fibrin [16]. The study was able to show that the resulting hydrogels could be employed in customizable tissue and organ 3D constructs. Human umbilical vein endothelial cells (HUVECs) were also co-cultured with fibrinogen as a supporting structure for attachment and elongation in a study by Sriphutkiat et al. [288]. They combined GelMA with fibrinogen to enhance bio-ink printability, since the dual crosslinking capacity of GelMA-fibrin was shown to provide a more robust and stable cell-laden construct. Enhanced stability may be due to the formation of an interpenetrating polymer network [288].

##### Silk

Silk, a fibrous insoluble protein, is produced by arachnids and myriapods, such as spiders and silkworms [289]. Silk chains are comprised of block polymer-like alternating hydrophilic and hydrophobic regions, giving the material amphiphilic characteristics and the capability to form semi-crystalline structures through hydrophobic interactions and crosslinking. Due to the hierarchical self-organization of silk, a variety of processing techniques have generated different forms of silk (e.g., fibers, solids, hydrogels, threads and sutures, etc.), which combine favorable strength, elasticity and hypoallergenic properties [289].

Among the available natural hydrogels, silk-based hydrogels are proposed as a promising biomaterial for developing tissue grafts that can be used in tissue engineering and regenerative medicine [290]. These silk scaffolds have been successfully used in the bioengineering of tissues [291], wound healing [292], bone regeneration [293], cartilage repair and regeneration [294], controlled drug release [295] and the 3D bioprinting of cellularized structures [38,39,40,296,297,298].

**Figure 8:**
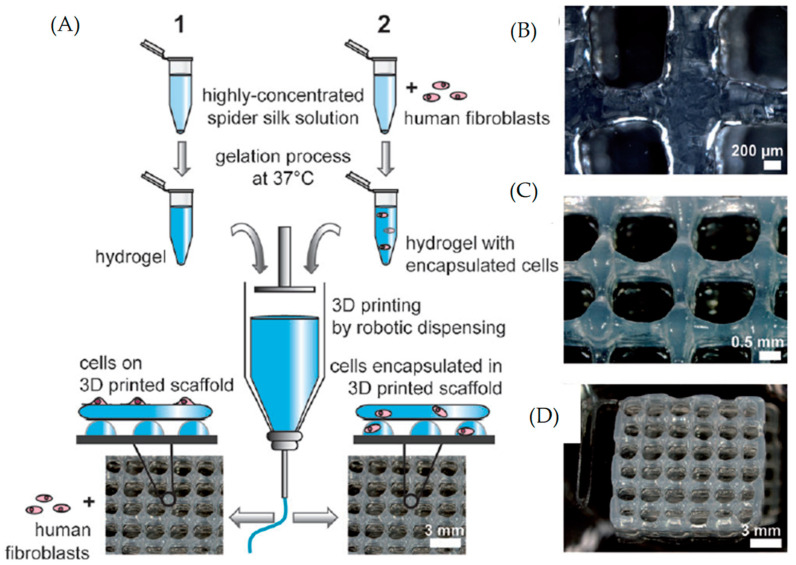
Assessment of the potential of recombinant spider silk protein as a bio-ink for 3D bioprinting by robotic dispensing: (**A**) 3D printing schematic. Cells are either seeded on the scaffold (1) or encapsulated during the printing process (2). (**B**) Stereo-microscopy and digital images of two-layer scaffold(C16) and (**C**,**D**) eight-layer silk protein bioprinted scaffolds (reprinted from Schacht et al., 2015 [299], with the permission of John Wiley & Sons Inc., published under license. Copyright © 2022 WILEY-VCH Verlag GmbH & Co. KGaA).

In the study by Bandyopadhyay and Mandal, a novel silk-based bio-ink was employed [38]. This bio-ink was composed of silk, fibroin and gelatin and was characterized by high print fidelity and shear-thinning properties. The silk-based bio-ink could facilitate the fabrication of a 3D bioprinted meniscus scaffold (laden with meniscus fibrochondrocytes) that could biomimic the internal and bulk architecture of the menisci. According to the study, the use of this novel silk-based bio-ink did not negatively affect the phenotype or the proliferation of the fibrochondrocyte cells seeded on the scaffolds, with observed improvements in glycosaminoglycan and collagen synthesis. A similar observation was also reported in the study by Rodriguez et al. [39], who showed that silk-based bio-inks could be used in complex soft tissue reconstruction and retained their structural integrity under physiological conditions, for the promotion of cellular infiltration and tissue integration. In the study, silk-based bio-inks were developed using gelatin as a bulk material and performing physical crosslinking with glycerol. It was also demonstrated that the silk-based bio-ink was biocompatible and promoted cellular infiltration and tissue integration. In a recent paper by Zheng et al., a silk-based hydrogel system in which silk gelation via β-sheet structure formation was controlled, using low molecular weight (LMW) polyethylene glycol (PEG), for enhanced hydrogel lubricity, was developed [41]. This silk-based hydrogel system contained PEG, and both the gelation time and mechanical properties were determined by variations in the PEG and silk concentrations. The study was able to show that human bone marrow mesenchymal stem cells in the silk-based hydrogel system maintained their viability and the cell-loaded constructs for (at least) 12 weeks. The study also showed that a positive correlation existed between increasing silk concentrations and cell growth. Further investigations showed that subcutaneous implantation of the silk-based bio-ink of 7.5% *w/v* in mice did not negatively affect cell viability, with the cells shown to survive and proliferate in the silk-based bio-ink for a minimum of 6 weeks after implantation. Similarly, silk–collagen composite hydrogels have been investigated for suitability for mesenchymal stem cell preconditioning and myocardial regeneration via cardiac patch development [296]. The study reinforced the significance of silk in hydrogels, given that silk–collagen composites presented improved cell survival within the fabricated scaffolds. Improvements in fine-tuning of silk-based bio-inks with respect to cell-material interactions were demonstrated by Schacht et al. [299]. In this study, recombinant spider silk protein was evaluated to assess its potential as a bio-ink. The study was able to show that when used as a bio-ink together with a cell attachment motif for scaffold fabrication, the silk protein supported the adhesion and proliferation of cells over a period of one week in spider silk scaffolds (Figure 8).

Table 4 provides some examples of protein-based bio-inks hydrogels used for 3D biofabrication of cellularized structures.

#### 3.3.2. Polysaccharide-Based Bio-Inks

##### Alginate

Alginate is a water-soluble polysaccharide of natural origin, derived from alginic acid, obtained from the cell walls of different species of brown seaweed (algae class *Phaeophyceae*). It is an anionic copolymer composed of β-D-mannuronic acid (M) and α-L-guluronic acid (G) residues, linked together with α-(1→4) glycosidic linkages. The proportion and distribution of these two monomers are decisive for a wide expansion of the physicochemical properties of alginate. Ordinarily, the blocks consist of three different forms of polymer blocks: consecutive G-residues, M-residues and alternating MG-residues [304]. Its chemical composition varies between different species and different parts of the algae [305].

The addition of a divalent cation causes the formation of an insoluble hydrogel. The reagent generally used for crosslinking is calcium dichloride (CaCl_2_), as well as other chelators (e.g., sodium citrate, ethylenediaminetetraacetic acid). Alginate crosslinking is mediated by ionic forces and is entirely reversible by chelation of the previously applied divalent cations [305]. Alginate is employed frequently in regenerative medicine and tissue engineering applications due to its ease of forming a hydrogel [304,306]. Due to this ease, alginate-based hydrogels are popular materials for creating microencapsulation of cells [307], wound healing [308], drug and cell delivery [309], regeneration of the nucleus pulposus [310] and bioprinting of various structures [47,153,188,207].

According to Axpe and Oyen, alginate is easy to print, handle and extrude while protecting the encapsulated cells; however, mechanical and rheological issues may limit its direct use in 3D bioprinting, with hybrid hydrogels proposed as a strategy to offset existing limitations [311]. For instance, the use of the hybrid hydrogel of sodium alginate with carboxymethyl cellulose (CMC) has been reported to present enhanced potential for application in 3D bioprinting processes, as performed tests showed favorable shape fidelity. Furthermore, employing composites in the fabrication of 3D scaffolds containing pancreatic cancer cells produced capability of retaining nearly 90% cell viability after 23 days [43]. In another study, hydrogel nanocomposite inks were investigated in terms of printing a 3D scaffold for enhanced biocompatibility and processability [45]. In the study by Olate-Moya et al., photo-crosslinkable, modified alginate was bioconjugated with chondroitin sulfate and gelatin. Graphene oxide nanofiller was used to enhance cell proliferation, printability and the fabrication of a suitable cartilage extracellular matrix [45]. Further enhancements of the cell proliferation capability of alginate-based bio-inks have also been investigated in the literature [23]. In the study by Jiang et al., a composite alginate-based hydrogel (alginate-gelatin) was investigated as a cell-laden bio-ink to 3D-bioprint in vitro breast tumor models [23]. The study was able to show that 3D bioprinted constructs were mechanically stiffer as the concentration of alginate increased and gelatin decreased. Moreover, this led to fewer cell-adhesion moieties and less viable multicellular tumor spheroids. Further increments in cell proliferation may be achieved via the introduction of a so-called overlay [32]. A study by Neufurth et al. showed the overlay of agarose and the calcium salt of polyphosphate. The resulting [polyP·Ca^2+^-complex] was incorporated into the alginate/gelatin/SaOS-2 cell scaffold, and its effect on cell proliferation was assessed. The study was able to show that the introduction of this overlay led to an increase in cell proliferation. Additionally, the mechanical properties of the cell-containing scaffold were observed to be enhanced with the introduction of 100 μm of polyP·Ca^2+^-complex, leading to an increase in Young’s modulus from 13–14 to ~22 kPa [32].

##### Hyaluronic Acid

Hyaluronic acid (HyalA), a polysaccharide of linear, unbranched structure, can be found naturally in the ECM of cartilage and synovial fluid. It is a major constituent of glycosaminoglycans and cartilage. Naturally, HyalA provides joint protection by boosting the viscosity of the synovial fluid and making joint cartilage more flexible. HyalA is an anionic copolymer characterized by molecular weights ranging from 10^3^ kDa to 10^4^ kDa and a chemical structure that consists of β-D-glucuronic acid and N-acetyl-β-D-glucosamine, linked by alternate glycosidic bonds (1→4) and (1→3) [312].

**Figure 9:**
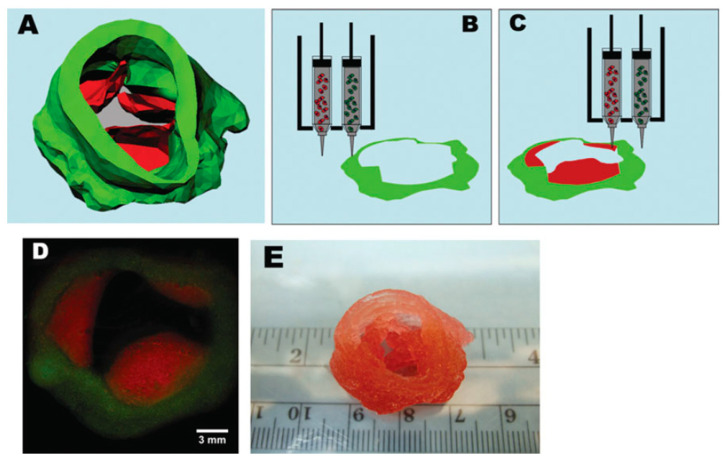
Bioprinting of aortic valve conduit: (**A**) aortic valve model based on micro-CT images. The root (green) and leaflet (red) regions were identified and rendered separately as 3D geometries, saved in STL format; (**B**,**C**) schematic illustrations of the bioprinting process with two different cell types and syringes; (**B**) root region of first layer of hydrogel embedded with SMC; (**C**) leaflet region of first layer obtained from VIC hydrogel; (**D**) fluorescent image of first two layers of aortic valve conduit; SMC for valve root shown in green and VIC for valve in red; (**E**) 3D bioprinted aortic valve conduit (Reprinted from Duan et al., 2013 [321], with the permission of John Wiley & Sons Inc., published under license. Copyright © 2022 Wiley Periodicals Inc.).

Regarding its mechanical properties, a single HyalA molecule shows viscoelasticity dependent on the pH and ionic strength present within its environment [313]. To improve its mechanical properties and form a robust biomaterial, HyalA can be chemically modified with a myriad of functional groups. The modified HyalA can be crosslinked to produce hydrogel that can be loaded with cells or other biomolecules. Different HyalA hydrogels were formed by photo-crosslinking of methacrylate groups incorporated into the HyalA chains. These groups, when subjected to ultraviolet irradiation, can undergo free radical polymerization, forming soft hydrogels [314]. The modified HyalA hydrogels have shown great potential in both tissue engineering and regenerative medicine applications, such as cutaneous and corneal wound healing [315,316], bone and cartilage repair [317,318], spinal cord injury repair [319] and generation of tumor models [314], as well as the 3D bioprinting of cellularized structures [53,211]. The properties of HyalA may also be fine-tuned via chemical modification [52]. The use of HyalA in 3D bioprinting is, however, limited by inherent difficulties associated with the fabrication of a controllable structure with desired shape and porosity. Several strategies have therefore been explored in order to utilize HyalA as a bio-ink for biofabrication. The study by Noh et al. [33] mentions a HyalA-based hydrogel composed of HyalA, hydroxyethyl acrylate and gelatin-methacryloyl, prepared with an intention to be used as a bio-ink. The resulting HyalA-based hydrogel could be effectively employed as a bio-ink, since when employed to fabricate lattice construct forms, no negative effects on embedded bone cell viability were observed. Another HyalA-based hydrogel that incorporated the thiol-modified HyalA and polyethylene glycol diacrylate was also prepared and investigated [320]. This new HyalA-based hydrogel was characterized by favorable gelation speed (within 1 day), with the resulting hydrogel characterized by a shear modulus that increased proportionally to the increase in the concentration of polyethylene glycol diacrylate. Stiffness of the resulting hydrogel depended on the availability of HyalA-thiols, while the addition of polyethylene glycol diacrylate facilitated a decrease in the steady-state stiffness post-gelation, in a dose-dependent manner. The study was able to show that the HyalA-based bio-ink composed of thiol-modified HyalA and polyethylene glycol diacrylate had cell-adhesive properties and could be tuned for enhanced cell adhesion and morphology. The possibility of utilizing a HyalA-based bio-ink in the fabrication of a tubular construct was demonstrated in the work by Skardal et al. [34]. Skardal et al. were able to show the development of a HyalA-based hydrogel containing methacrylated ethanolamide (GE-MA), which is a derivative of gelatin. The hydrogel was developed by utilizing a photo-crosslinking strategy, leading to a hydrogel with extrudable gel-like properties. The study demonstrated that the HyalA-based hydrogel was biocompatible and could support HepG2 C3A, Int-407 and NIH 3T3 cell attachment and proliferation in vitro. Furthermore, when the hydrogel was employed as a bio-ink in the fabrication of a tubular construct, the cells encapsulated in the construct retained their viability in cultures. Crucially, the construct was able to accurately mimic a naturally secreted extracellular matrix. The capacity of utilizing HyalA-based bio-ink in the fabrication of complex and important constructs such as the human leaflet trileaflet heart valve was also demonstrated in the study by Duan et al. [321] (Figure 9). In the study, a HyalA-based bio-ink containing HyalA and gelatin gels was prepared, and then human aortic valve interstitial cells were seeded on the scaffold. The HyalA-based bio-ink was able to show high cellular survival; moreover, remodeling activity was also observed after 7 days of culturing, thus further highlighting the utility of the HyalA-based bio-ink in promoting cell differentiation and mimicking naturally extracellular matrixes [322]. In a study by Lee et al., the HyalA-based bio-ink composed of HyalA and sodium alginate showed improved cell proliferation rate (~70% higher than for sodium alginate lone). Furthermore, the CaCl_2_ crosslinking did not lead to any significant shrinkage of the constructs, with integrity also maintained in culture [322].

##### Chitosan

Chitosan is a chitin-derived biopolymer, which can be found in crustacean and invertebrate exoskeletons or fungi. Chitosan in a partially or fully deacetylated chitin, included in the amino-polysaccharide group with molecular weights between 50 kDa and 2000 kDa. Chitosans’ degree of deacetylation can range from 40% to 98%. It is a cationic heteropolymer composed of linear β-D-glucosamine (GlcN) chains and units of N-acetyl-β-D-glucosamine (GlcNAc) together by β-(1→4) glycosidic linkages [323]. Chitosan is insoluble in neutral and basic conditions, but solutions of chitosan can be obtained in an aqueous acidic medium that charges amino groups positively, thereby overcoming associative forces between chains. An aqueous solution of chitosan subjected to alkalization to a pH above 6.2 causes precipitation of hydrogel due to the presence of ionic forces. A mixture of chitosan and glycerol-phosphate was already studied to synthesize a hydrogel behaving like a liquid at physiological pH and room temperature and was able to form a gel at a physiological temperature [324,325].

Chitosan-based hydrogels have already been studied in many biomedical applications, mainly as wound dressings and transdermal patches [326], drug delivery systems [327], skin and bone regeneration [328,329,330], cartilage tissue engineering [331] and blood vessel embolization [332,333], as well as for cell encapsulation or the 3D bioprinting of cellularized structures [24,46,58,59,60,61,62,334].

**Figure 10:**
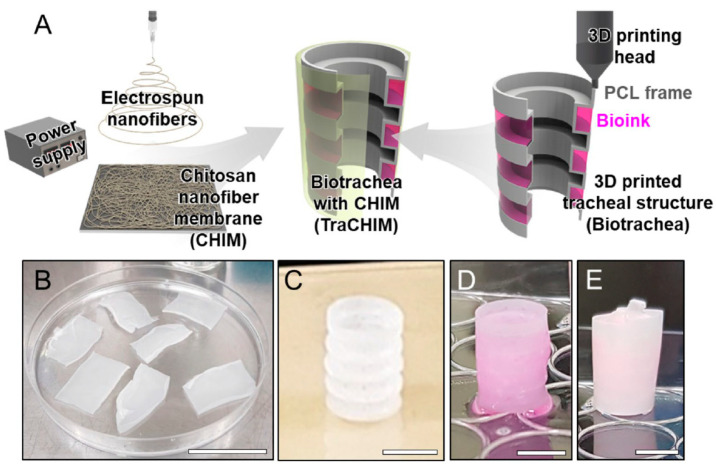
(**A**) 3D-bioprinted tracheal structure (biotrachea) obtained with an electrospun chitosan-based nanofiber membrane (CHIM). Figures (**B**–**E**) denote the CHIM, 3D-printed PCL frame, biotrachea, and the biotrachea with CHIM, respectively (reprinted from Kim et al., 2021 [337]. Copyright © 2022 Springer Nature under the terms of the Creative Commons Attribution 4.0 International License).

Having established that chitosan constitutes a promising biomaterial candidate for biological applications, its weak mechanical performance has so far inhibited its application in hard tissue engineering [335]. To resolve this challenge, researchers have investigated several chitosan modification techniques, to enhance the properties of chitosan-based bio-inks [24]. He et al. achieved chitosan modification using ethylenediaminetetraacetic acid (EDTA) for the provision of carboxyl groups prior to physical crosslinking using calcium for enhanced strength of the resulting construct. The study was able to demonstrate that improved chitosan-based bio-ink promoted cell attachment and chondrogenic gene expression in chondrocytes. Notably, Roehm et al. also stated that chitosan-based bio-inks had the potential to resolve issues associated with the bioprinting of cell-laden structures characterized by controlled spatial relations [24]. This assertion was shown by Roehm et al. via a demonstration of the functionality of utilizing a chitosan-based bio-ink composed of chitosan–gelatin to fabricate constructs while maintaining cell viability. Another study investigated a chitosan-based bio-ink prepared from chitosan, glycerophosphate and hydroxyethyl cellulose and embedded with cellulose nanocrystals (CNCs), to produce a nanocellulose/chitosan-based bio-ink [336]. The study demonstrated that the addition of CNCs to the bio-ink improved the viscosity of bio-inks containing cells (5 million cells/mL) and enhanced scaffolds’ mechanical properties. The CNCs were also shown to increase the osteogenesis of MC3T3-E1 cells enveloped in chitosan scaffolds. Moreover, extracellular matrix formation was observed when bio-ink contained chitosan. Another study by Ramesh et al. focused on the preparation of scaffolds using a thermo- and pH-responsive chitosan-based bio-ink composed of chitosan and glycerol phosphate [86]. This thermo- and pH-responsive chitosan-based bio-ink was shown to present antibacterial activity when formulated with zinc oxide nanoparticles, while also retaining the osteoconductivity of the chitosan-based bio-ink hydrogel. Chitosan-based bio-inks can be used to resolve limitations in cartilage reconstruction that characterize tracheal tissue engineering, such as the poor delivery of chondrocyte-laden components [337]. According to the study by Kim et al., a chitosan-based nanofiber membrane (Figure 10) composed of chitosan and polycaprolactone facilitates an improvement of the mechanical properties of chitosan but also demonstrates enhanced chondrogenic performance when used in the fabrication of a tissue-engineered trachea. Indeed, the implantation of the chitosan design to a tissue-engineered trachea in male rats showed an elevated number of chondrocytes within the implanted model when compared to the control group without the proposed membrane [337].

##### Cellulose

Cellulose is the most widely used and most abundant biopolymer in nature and is produced by some algae, fungi and bacteria. It is a linear homopolymer that possesses a 3D matrix that is responsible for its favorable tensile properties and crystalline form [338,339,340]. Cellulose is built from repeating cellobiose units, specifically two β-D-anhydroglucopyranose units linked by β-(1→4) glycosidic linkages. This arrangement leads to a ribbon structure, stabilized by intramolecular hydrogen bonds. Numerous intermolecular hydrogen bonds combine molecules into an elementary microfibrillary structure, which in turn combine to form fibers that exhibit a crystalline structure [339]. It is this crystallinity that gives the plant walls their rigidity and insolubility in water [339].

To improve cellulose water solubility, various cellulose derivatives were synthesized, mostly by etherification of the hydroxyl groups on anhydroglucose units of cellulose [341]. Hence, the most widely used are hydroxypropylmethylcellulose (HPMC) and carboxymethylcellulose (CMC), which belong to the large family of cellulose ethers that includes, among others, methylcellulose (MC) and hydroxyethylcellulose (HEC) [341]. Cellulose and its derivatives have been widely used in the pharmaceutical industry due to their ability to swell and their high compatibility, which makes them suitable for drug delivery in oral tablet and capsule formulations [342,343,344]. Additionally, cellulose derivatives may be able to form hydrogels that exhibit favorable biological and rheological properties for biomedical applications [345,346], mainly wound dressing, transdermal patches [326,347], ophthalmic preparations [348] and cartilage tissue engineering [349,350], as well as the 3D bioprinting of cellularized structures [21,43,49,63,64,65,66].

**Figure 11:**
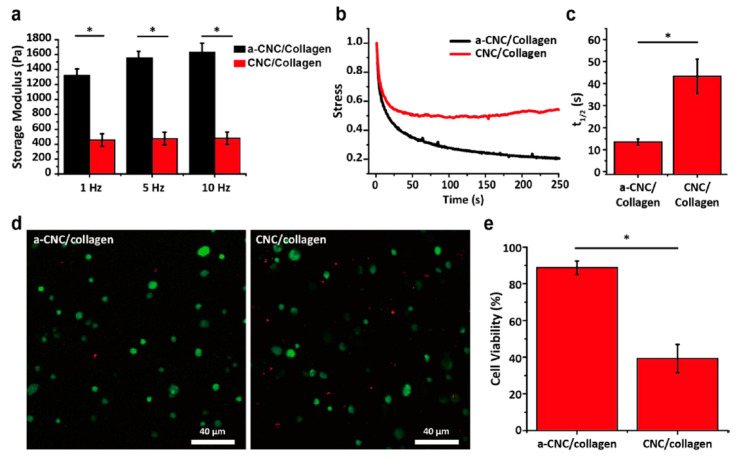
(**a**) Storage modulus of a-CNC/collagen and CNC/collagen hydrogels at frequencies of 1, 5 and 10 Hz; (**b**) representative relaxation stress curves of the hydrogels at a strain of 1%, which was normalized to the initial stress; (**c**) stress-relaxation time of the hydrogels when the stress dropped to half of its initial values; (**d**) representative live/dead staining images of the MSCs encapsulated in the hydrogels post-injection (scale bar 100 μm); (**e**) quantification of cell viability from the live/dead staining (reprinted from Zhang et al., 2020 [21], with the permission of American Chemical Society, published under license. Copyright © 2022 American Chemical Society). “*” highlights the statistical significance of the differences.

Recognizing the potential of cellulose as a biomaterial for bioprinting due to its biocompatibility properties, several researchers have sought to explore approaches to improve the inherent limitations of cellulose due to its poor mechanical properties [351]. For instance, in the study undertaken by Habib et al., a cellulose-based bio-ink composed of sodium alginate with carboxymethyl cellulose was developed [351]. Chemical modification of cellulose to produce carboxymethyl cellulose involved using carboxymethyl groups (-CH_2_COOH) to replace the hydroxyl group present in the glucopyranose chains of cellulose [352]. This bio-ink was shown to demonstrate good printability and shape fidelity. Notably, when a cellulose-based bio-ink embedded with cells was used in the fabrication of scaffold structures, a high cell viability of 86% was recorded after 23 days. Another study also investigated a cellulose-based bio-ink composed of carboxymethyl cellulose, hydroxyapatite (HA), gelatin and chitosan to develop a bio-ink useful for fabricating scaffolds of favorable mechanical properties. The presence of gelatin served to promote cell growth and proliferation, with the interaction between the chitosan and the carboxymethyl cellulose shown to promote good hydrogel bone tissue infiltration. A cellulose-based bio-ink that incorporated sodium alginate was also developed and investigated by Gospodinova et al. [353]. In the study, a cellulose-based bio-ink of hydroxyethylcellulose blended with various concentrations of sodium alginate was embedded with HeLa cell lines sourced from cervical cancer cells. The study was able to establish an inverse correlation between sodium alginate and cell viability. When the bio-ink was employed in the printing of a cervical tumor model, it was observed that bio-inks containing 1% and 2.5% of sodium alginate did not present negative effects on cell viability, even after residence times of up to 90 min were imposed prior to bio-ink extrusion. The potential of utilizing cellulose-based bio-ink in stem cell therapy for the regeneration of articular cartilage while retaining high cell viability was also investigated by Zhang et al. [21]. In the study, a cellulose-based hydrogel composed of surface-modified cellulose nanocrystals (CNCs) and collagen hydrogel (a-CNC/collagen), crosslinked rapidly with dynamic Schiff base bonds, was obtained. This novel material exhibited shear-thinning and self-healing behaviors. Moreover, it showed higher elastic modulus compared to the cellulose-based hydrogel in the absence of dynamic Schiff base bonds, as shown in Figure 11. Additionally, a-CNC/collagen hydrogel was investigated as a platform for mesenchymal stem cell (MSC) delivery, and the results proved a high cell viability even after extrusion in vitro.

##### Agarose

Agarose is a natural-based polysaccharide obtained from agar-agar, which is extracted from red seaweed (specifically, algae class *Rhodophyceae*). It is a non-ionic and linear copolymer composed of repeating units of β-D-galactose and 3,6-anhydro-α-L-galactopyranose residues, linked together by alternating glycosidic linkages (1→4) and (1→3) [354]. Agarose is widely used to study the thermo-reversible gelation of polysaccharides. There are two key factors impacting agarose hydrogel formation: temperature and concentration. At high-temperature, agarose chains in solution exhibit a random coil structure, but when the temperature is lowered, they form single or double helical structures, which then aggregate to form a bundle and later a gel [354,355,356,357]. Agarose proneness to form hydrogels without the presence of toxic crosslinking agents and catalysts is high and even further enhances its biocompatibility [358,359]. These agarose-based hydrogels have been investigated and applied in biomedical applications as self-healing materials [359], for cell culture [360], cartilage tissue engineering [361], drug release [362] and 3D bioprinting of cellularized structures [32,46,67,68,69,70].

In recognition of the potential of using agarose in a biomaterial 3D bioprinting, Gu et al. [69] developed a novel agarose-based bio-ink. This bio-ink was composed of carboxylated agarose (CA) and native agarose (NA) (composed of 7.8% *w*/*v* CA and 0.2% *w*/*v* NA solids). The study demonstrated that sol-gel transition was exhibited by the agarose-based bio-ink at a physiological temperature of 37 °C, with the structures produced using the bio-ink shown to be stable in the temperature range of 4–37 °C. This agarose-based bio-ink was also shown to support a high density of cells (i.e., 30 million/mL) without loss of printability. In another study, an agarose-based bio-ink containing agarose and alginate, prepared as 5% *w*/*v* (mass ratio 3:2 agarose to alginate) [68], was developed and assessed. The study was able to show that the printability and rheology of the agarose-based bio-ink were comparable to Pluronic, a synthetic poloxamer that is widely used in tissue engineering [363]. The agarose-based bio-ink was also shown to demonstrate excellent cell viability after 28 days, with 70% cell survival reported on day 28. Furthermore, an agarose-based bio-ink was also shown to be excellent in the fabrication of 3D tissues that retained induced pluripotent stem cells (iPSCs) [46]. In the study by Gu et al., agarose-containing bio-ink composed of agarose, alginate and carboxymethyl-chitosan of 1.5, 5 and 5% *w*/*v*, respectively, and crosslinked with calcium chloride was proliferated with iPSCs. The resulting bio-ink was able to overcome established difficulties associated with iPSC differentiation and maintenance in printed scaffolds, with the agarose component in the composite bio-ink providing the essential rheological properties required for printing. The capacity of utilizing agarose-based bio-inks to facilitate cell-induced vascularization was also demonstrated in the study by Kreimendahl et al. [364]. Their study showed that the formation of capillary networks by human umbilical vein endothelial cells and human dermal fibroblasts in a blend of agarose and type I collagen was promoted. Moreover, they reported that printing resolution was not limited by the addition of collagen, with the bio-ink capable of promoting cell-induced vascularization capability. It is also possible to chemically modify the chemistry of agarose via the introduction of carboxylic acid groups on the polysaccharide backbone to produce carboxylated agarose, for improved mechanical properties [253]. The use of such agarose-based bio-ink of carboxylated agarose was demonstrated in the study by Forget et al., where carboxylated agarose was employed in the bioprinting of human mesenchymal stem cells, with a 95% cell survival rate reported [365]. Similarly, the use of such agarose-based bio-ink of carboxylated agarose was shown to facilitate stiff 5–10 mm constructs in the absence of additional support materials [69].

##### Carrageenan

**Figure 12:**
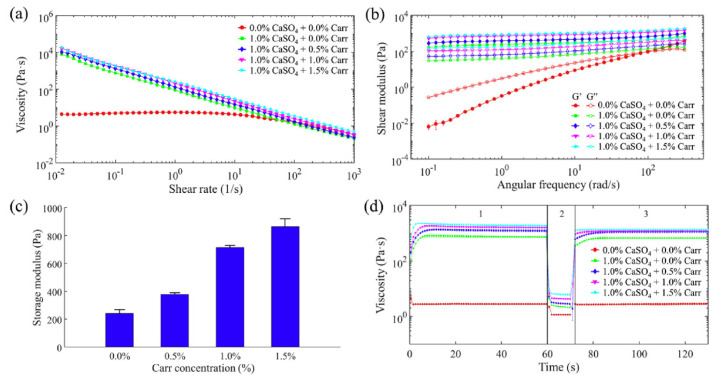
Rheological properties of a carrageenan-based bio-ink: (**a**) viscosity of the Alg-Carr-CaSO_4_ as a function of shear rate (0.01–1000 s^−1^); (**b**) shear modulus of the Alg-Carr-CaSO_4_ composite as a function of angular frequency; (**c**) increasing storage modulus as the carrageenan concentration increases; (**d**) the variation of viscosity at various hydrogel compositions (reprinted from Kim et al., 2019 [377], with the permission of Elsevier Ltd., published under license. Copyright © 2022 Elsevier Ltd.).

Carrageenan is a high-molecular-weight sulfated polysaccharide, produced by red seaweed of the algae class *Rhodophyceae*. It is a linear anionic polymer composed of repeating units of β-D-galactopyranose and 3,6-anhydro-α-D-galactopyranose and linked by α-(1→3) and β-(1→4) glycosidic linkages [366]. The prominent feature of carrageenan is its diversity, depending on the algae source and extraction methods. Three main types of carrageenan can be obtained with similar chemical structure characteristics, namely kappa (κ), iota (ι) and lambda (λ) [367]. However, the level of the sulfate ester of each type strongly influences the gelation and the solubility temperature, as well as the gel strength [368]. With large and highly flexible molecules, forming a spiral structure, κ-carrageenan shows thermoreversible hydrogel-forming ability. It forms the strongest hydrogel with potassium ions but also shows gelation under salt-free conditions [367]. Carrageenan has shown several potential biological and pharmaceutical applications, such as controlled drug release due to favorable biocompatibility [369,370,371]. Other biomedical applications of carrageenan-based hydrogels include tissue engineering [372], skin regeneration [373], wound healing [374], cartilage scaffold [375] and the 3D bioprinting of cellularized structures [71,72,73]. Crucially, carrageenan-based bio-inks have been identified as being able to circumvent the existing limitations of bio-ink-based bioprinting [71], such as the shear stress imposed on the cells and the poor ability of bio-inks to maintain complex tissue structures [71]. In this regard, Lim et al. synthesized a methacrylated kappa-carrageenan (MA-κ-CA) bio-ink through dual crosslinking via ionic and ultraviolet crosslinking [71]. The MA-κ-CA bio-ink also contained mouse-sourced fibroblast (i.e., NIH-3T3) cells. This bio-ink was shown to present favorable biocompatibility, biodegradability and shear-thinning properties, with the cell-laden MA-κ-CA shown to be able to fabricate constructs characterized by an enhanced shape retention capability. The carrageenan-based bio-inks may also be used in the fabrication of materials that have swelling resistance, as illustrated in the study by Jiang et al. [376]. They obtained an ink composed of polyvinyl alcohol (PVA) and κ-carrageenan via freezing and thawing processes to induce a physically crosslinked network formation. The study showed that in the carrageenan-based bio-inks, cells demonstrated the capacity for surface attachment and were capable of also stretching into the spaces in the grid architectures, for the provision of ideal microenvironments for cell culture. Another study designed a bio-ink composed of alginate and carrageenan [377]. The study was based on CaSO_4_ as the crosslinking agent, to produce the bio-ink designated as Alg-Carr-CaSO_4_. The results show that the rheological and mechanical properties of hydrogel improved as the concentration of carrageenan in the composite hydrogels increased, with a carrageenan bio-ink of concentration of 1.5% *w/w* shown to present the best properties (Figure 12). Additionally, cell viability seeded on the composite scaffolds was evaluated using rabbit adipose-derived mesenchymal stem cells [377].

Table 4 provides some examples of polysaccharide-based bio-inks carrageenan-based hydrogels used for 3D bioprinting of cellularized structures and organs.

##### 3.3.3. dECM-Based Bio-Inks

In some cases, hydrogel-based bio-inks are combined with dECM to produce dECM-based bio-inks for enhanced biocompatibility as a basis for mimicking 3D bioprinted constructs [30]. This is because dECM-based bio-inks have the capacity to promote cell–matrix interactions and organ (or tissue)-specific differentiation processes for the recreation of original cellular functions [378]. Such bio-inks are equipped with cell surface receptors in their adhesion sites and have the capacity for the preservation of normal tissue function due to their capacity to mimic tissue-specific mechanical and biochemical properties [379,380]. Due to these benefits, dECM-based bio-ink formulations now constitute an emerging field in tissue engineering [197].

Hydrogels containing dECM retain the ECM function, with crucial structural characteristics and stimulatory properties [381]. Indeed, these dECM-based bio-inks have generated significant interest due to promoting re-cellularization for the production of functional tissues or organs while also encouraging cell differentiation and cell proliferation, as highlighted in the literature [29,30]. In the study by Ali et al. [29], dECM was derived from porcine whole kidneys, and the photo-crosslinkable ECM hydrogel was produced via the main steps of decellularization, pepsin-mediated solubilization, and chemical modification via methacrylation of the kidney ECM-derived hydrogel. The dECM based hydrogel (3% *w*/*v*) presented a high mechanical and structural stability, with a modulus of 4405 ± 277 Pa. Notably, it also facilitated the proliferation of human kidney cells, thus highlighting the unique benefit of this bio-ink type. In other studies, Kim et al. [382] employed dECM bio-ink derived from the pancreas and stem cell-derived dECM in the development of a 3D islet construct and corneal construct, respectively. In both cases, the appropriate mechanical properties of the dECM bio-inks for 3D bioprinting technology and their ability to enhance tissue-specific differentiation compared with conventional bio-inks were demonstrated.

Table 4 provides some examples of dECM-based hydrogels used for 3D bioprinting of cellularized structures and organs.

#### 3.3.4. Multi-Component Bio-Inks

Section 3.3 includes a discussion related to so-called multi-component bio-inks. These multi-component bio-inks, as the name implies, are bio-inks composed of multiple biomaterials, cells, additive materials or biomolecules [383]. These multi-component bio-inks seek to circumvent the limitations of conventional ‘mono’ bio-inks, such as the inability to satisfy all the mechanical and functional requirements necessary to obtain biomimetic tissue-like models [383]. Thus the biomaterials in multi-component bio-inks complement one another by serving as supplementing elements that enhance the formation of more complex tissue constructs [253]. Multi-component bio-inks are particularly relevant when employed as hydrogels, since simple hydrogels are typically characterized by poor mechanical properties. The development of such multi-component bio-inks was highlighted in the study of Pitton et al. [384], in which multi-component bio-inks, based on the combination of natural biomaterials of pectin and TEMPO-oxidized cellulose nanofibers (TOCNFs), were prepared as an approach to optimize the printability and stability of cell-laden inks. The study was able to determine that the multi-component bio-ink containing optimal TOCNFs and pectin concentrations of 1% *w*/*v* and 2.5% *w*/*v* improved viscosity while maintaining shear thinning behavior and cell viability. It was also determined that the resulting printed scaffolds had an elastic modulus of E = 1.8 ± 0.2 kPa, while cell viability was >80%. In another study by Markstedt et al., [63], a multi-component bio-ink containing of nanofibrillated cellulose and alginate was developed as an approach to benefit from the unique shear thinning and fast crosslinking properties of the biomaterials. The study was able to show that the resulting bio-ink showed a cell viability up to 86% after 7 days, when laden with human chondrocytes. Similarly, Chung et al. investigated the bioprinted, multi-component scaffold containing alginate and gelatin to achieve the combined benefits of improved mechanical properties and enhanced cell proliferation [385]. Alginate has also been previously combined with materials, e.g., with fibrin, to enhance the interaction of the bio-ink with cells [386]. Limitations and advantages of natural-based bio-inks are summarized in Table 5.

A review of the existing literature also highlights the commercial status of some of these natural-based bio-inks. For instance, BIOGELX currently sells bio-inks such as Bio-gelxTM-INK-Arg-Gly-Asp (RGD) and Bio-gelxTM-INK-GFOGER, which are protein-based bio-inks functionalized with fibronectin and collagen, respectively [387]. Another company, Gelomics, is also reported to be involved in the sale of GelMA-Bovine and GelMA-Porcine bio-inks, which are based on bovine and porcine gelatin [387]. Other companies, such as Advanced Biomatrix, Corning and Brinterbio-inks, are also involved in the commercial production of collagen I, Lifeink^®^ 240 and Corning^®^ PuraMa-trix^™^ bio-inks, which are based on collagen protein [387]. Polysaccharide-based bio-inks are also commercially available for sale as CELLINK Bio-ink by Cellink, composed of alginate and highly hydrated cellulose nanofibrils [388]. Similarly, another company, UPM Biomedicals, also produces a natural-based bio-ink from nanofibrillar cellulose [389]. Recognizing the importance of these natural-based bio-inks to tissue engineering, it is anticipated that more work will be undertaken in the development of new hydrogels based on naturally derived polymers.

**Table 5:** Advantages and disadvantages of natural bio-inks.

Natural-Based Bio-Inks	Advantages	Disadvantages	References
Collagen	This hydrogel may enhance cell function/attachment. This is because collagen can interact with elastin fibers for the provision of a recoil to the extracellular matrix and fibronectin.	The hydrogel product is characterized by poor mechanical properties. The rapid biodegradation rate may also limit its utility. The hydrogel may also have challenges such as thrombogenicity, contamination, and source and batch variability.	[178,390,391]
Gelatin	The hydrogel possesses excellent biocompatibilities and nonimmunogenicities.	The bio-ink is characterized by its poor mechanical properties and short degradation times, thus limiting its applicability in the production of hydrogels and stable scaffolds.	[392,393]
Fibrin	This hydrogel has excellent biocompatibility and biodegradation properties.	The hydrogel is characterized by weak mechanical properties.	[285]
Silk	Silk-based hydrogels have excellent printability and high resolution. Additionally, cell viability can be maintained.	The bio-ink has poor mechanical properties and unfavorable swelling behavior.	[178,394]
Alginate	This bio-ink can undergo gelation under mild conditions using non-toxic reactants such as via substitution of the sodium ions from the guluronic acids with the divalent cations. The bio-ink also has favorable properties of non-toxicity, biocompatibility, biodegradability and hydrophilicity.	This bio-ink may have poor stability and poor mechanical and barrier properties. The bio-ink has heat treatment instability.	[395,396]
Hyaluronic acid	The bio-ink has favorable properties of biocompatibility, inherent bifunctionality, non-immunogenicity, versatility and biodegradability.	The bio-ink is characterized by poor mechanical properties and rapid degradation. Degradation occurs via oxidative species and enzymatic degradation.	[397,398]
Chitosan	The bio-ink has favorable flexibility properties and is non-toxic.	The bio-ink has limitations associated with its poor stability, poor mechanical properties, and difficulty in pore size control.	[399]
Cellulose	The resulting construct may have favorable water retention and high cell viability after printing. The bio-ink also has favorable biocompatibility, reduced toxicity and high crystallinity. It also may easily form high tensile strength gels.	The bio-ink has poor dissolution and therefore has some applicational limitations.	[400,401]
Agarose	The bio-ink requires comparatively low gelation temperatures (i.e., 40 °C). The bio-ink also produces constructs with good shape fidelity.	Due to viscosity plugging limitations, agarose is not a frequent material choice for bioprinting procedures. Significant temperature control in microvalve printing is also required. Although constructs prepared using this bio-ink have good shape retention, the construct may be limited by brittleness issues.	[402,403,404]
Carrageenan	This bio-ink is characterized by an abundance of functional groups that presents opportunities for chemical modification and thus the enhancement of the physicochemical properties of the produced hydrogel. The bio-ink also possesses the favorable properties of biocompatibility, hemostatic ability, and antioxidant and immunomodulatory properties. The bio-ink also has good gelation properties.	This bio-ink is limited by the uncontrollable exchange of ions as well as the potential to form a brittle hydrogel.	[405,406,407]
dECM-based bio-inks	The resulting construct from dECM is characterized by high cell viability and functionality.	This bio-ink may be limited by its higher cost compared to other natural-based bio-inks. This is because of the associated cost of the isolation/or quantification of ECM constituents.	[408]
Multi-component bio-inks	The limitations associated with single component hydrogel bio-inks, such as poor print fidelity and shape retention, poor biofunctionality and poor cell-instructive capacity, can be circumvented.	These bio-inks require precise control of the rheological properties of multicomponent bio-inks.	[409]

## 4. Recent Trends in Bioprinting and Bio-Inks

At the time of writing this manuscript, there was no “all-inclusive” source in the literature that contained all the information and discussions on naturally derived hydrogels synthesized from various natural sources, such as polysaccharides, proteins, etc., and their unique utility in biomedical applications.

Bioprinting has advanced rapidly over recent years due to engineering breakthroughs in 3D printing devices and technologies. More advanced bioprinting techniques are emerging, and new materials are being rapidly developed. Notable technology examples include multimaterial 3D biofabrication [410], volumetric bioprinting [411], and voxel-based [412] and co-axial 3D printing [413,414]. The revolution in bioprinting technologies came with new hydrogel bio-inks, made of natural materials, reminiscent of biological ECM [83], that constitute a scaffold on which the cells can attach, diffuse, interact, differentiate and proliferate. Future bio-inks will be easily printable and adjustable to the tissue they mimic. Living inks will be designed to guide tissue-scale self-organization of cells and their differentiation into specific tissues [415]. Recent design strategies are based on the combination of natural (e.g., collagen, gelatin, silk) and slightly modified hydrogels (e.g., Gel-MA) with decellularized body fluids and extracellular matrices, including plasma-rich [40,416] and dECM [417] bio-inks. Undoubtedly, more and more frequently, multi-component bio-inks are being introduced in research studies. The inclusion of additional materials makes it possible to obtain functional multicomponent-based bio-inks capable of improving the overall properties and rendering good functionality.

Recently, the concept of time has been introduced to 3D bioprinting as the fourth dimension, leading to 4D bioprinting. In 4D bioprinting, printed bioactive objects are programmed to undergo shape or functional changes according to the desired stimulation with time [418]. The best-known biomedical examples of 4D bioprinting include drug delivery systems and vessel formation. In the former, 4D bioprinting allows for the precise control of components’ spatial distribution, that can be programmed to shrink and swell to, e.g., release drugs in a specific place in the body. In the latter, 3D-printed networks are induced by the environmental factor to fold into tubes, mimicking vascular-like tissue constructs. Such 4D biofabrication processes should not have any negative effect on cell viability and should react to stimulation in a programmed manner, and the tubes should support cell survival and growth [419]. Shape-morphing behavior usually arises through assembling multiple materials of different swelling responses. As 3D bioprinting moves into the future, it is highly desirable to use several different materials in a single printing process. Certainly, with the upcoming printers, one will be able to produce an object containing multiple materials, including hydrogels, elastomers, metal, and even ceramics [420]. Multi-material bioprinting will be invaluable in comparison to conventional bioprinters for the fabrication of constructs that are inherently complex, heterocellular, and hierarchically arranged within an extra-cellular matrix, just like native tissues [421].

Most of the current technologies employing 3D bioprinting are based on extrusion printing, stereolithography, and laser-based methods. They make it possible to accurately control the spatial arrangement of cells and biomaterials through automated processes. However, these methods may have some disadvantages. For example, they sometimes fail to produce complex geometries, mimicking native tissues. To overcome these issues, a new strategy for 3D bioprinting—volumetric bioprinting (VBP)—has only recently been proposed. VBP is inspired by the principle of computed tomography. It assumes deflection of cell-friendly visible laser light onto a photosensitive hydrogel loaded with cells. Volumetric bioprinting allows the production of geometrically convoluted, centimeter-scale architecture in a relatively rapid manufacturing process. The object can be manufactured at once, rather than through successive deposition of material layers [411]. VBP enables easy scalability in rapid prototyping, paving the way to new applications in tissue engineering and regenerative medicine approaches.

Nowadays, it is well acknowledged that 3D printed hydrogel functionality depends significantly on its crosslinked structure. Gelation density has a direct influence on basic hydrogel properties, such as swelling, elasticity, mechanical strength, diffusion, permeability, and even cell viability and degradability [227]. In addition to using hydrogels to regenerate or reproduce organs or tissue, attention should be also focused on bio-ink design for hydrogel-based 3D bioprinting. Therefore, a hydrogel-based bio-ink should include several critical attributes and functionalities of hydrogels (e.g., printability, shape stability, functionality, degradability and biocompatibility with native tissue environment) [197].

Over the years, the research on bioprinting has been mainly focused on developing new printable materials and adapting the existing printing technologies to new approaches. Research labs have been outdistancing each other in creating a wide variety of living human tissue constructs. However, the biological phenomena involved have been barely touched. Careful consideration of the technologies employed in 3D bioprinting discussed within this paper shows that these technologies are influenced by several printing and bio-ink parameters. However, bioprinting has yet to find a set of these parameters that simultaneously enables a successful printing process and provides the highest cell viability.

## 5. Future Trends and Conclusions

By thoroughly exploring current trends, it is possible to speculate that future bioprinting will shift towards rationally designed cellular structures (organoids) that have a particular biological function, capable of treating a specific disease or studying the mechanism beyond it rather than mimicking the functionality of the whole organ. 3D biofabrication has enabled a transition from a 2D organ-on-a-chip to a multicellular in vitro 3D tissue construct that recreates native in vivo organs in the culture dish [422,423]. For now, bioprinting has been used, e.g., to fabricate kidney [89], brain, liver [424] and tumor [300]. Given the dependence of constructs’ cell differentiation, proliferation and mechanical characteristics on the bio-ink properties, it is anticipated that research into the utilization of biopolymers as natural polymers (protein-based polymers, e.g., collagen, fibrin, silk, etc.) and polysaccharide-based polymers (e.g., cellulose, agarose, alginate, etc.) in the development of bio-ink formulations will increase. This is because natural polymers are not only biocompatible but also have the advantage of promoting enhanced cell–material interaction when incorporated in polymeric solutions. In spite of this unique advantage of natural polymers in bio-ink formulations, there is a need to further enhance the dynamic interactions between natural tissues, cells and the environment [425]. Additionally, the development of bio-ink formulations using natural polymers may be limited by long cell cycle times (weeks to months), implying that long times may be required for the development of bio-inks for the fabrication of complex constructs or organs [426]. More work is also required to provide better tuning of the bio-ink signaling cues for the stimulation of cell differentiation and proliferation [426]. Further investigations in this research area are anticipated to promote strategies to optimize the mechanical, rheological and biological properties of the bio-ink to support the fabrication of larger and more complex constructs and vasculature. It is expected that more work will be undertaken in the field of integrating computational modeling in bioprinting approaches. This is because such computational modeling could be invaluable in tentatively predicting bio-ink printability, thus allowing hydrogels to encourage the transport of useful growth factors that provide the required cell differentiation and proliferation. These expectations were also echoed by Feinberg et al. [427], who stated that sustained research in this area will lead to the discovery of opportunities for improved 3D bioprinting, thus empowering favorable outcomes in the precision design of engineered tissues, organoids, and even complete organs. Indeed, the viability of complete organ fabrication was recently demonstrated by researchers at Lund University [428]. These researchers undertook the 3D bioprinting of human airways using a novel tissue-specific hybrid bio-ink composed of alginate enriched with decellularized extracellular matrix (dECM). The proof of concept showed that it was possible to fabricate human airways using bio-ink embedded with primary human airway epithelial progenitor and smooth muscle cells. The scaffold maintained their viability and differentiation in vitro for one month. This recent breakthrough in complete organ fabrication further supports the assertion that future approaches may involve the complete phase-out of the use of autologous grafts and organ transplants in lieu of the use of bioartificial constructs.

It is also anticipated that more research into so-called smart natural hydrogel-based bio-inks incorporating responsive moieties that facilitate responses to different environmental conditions such as pH, electric field, magnetic field, etc., will be undertaken [429]. The development of such smart bio-inks may provide a pathway for inducing transitional changes within the cell-laden matrix, thus enhancing utility in tissue engineering and sustained drug release [429,430]. This is because external factors such as injury and disease can lead to changes in the fabricated constructs from natural hydrogel-based bio-inks and therefore enable a targeted and time-dependent response [387]. It is acknowledged that although the natural hydrogel-based bio-inks are characterized by favorable biocompatibilities, there still exists a risk that the use of such natural hydrogels may induce an immune response. It is anticipated that future works will investigate approaches to mitigate this risk [387]. Additionally, we predict that future research will seek to enhance post-printing cellular proliferation via the development of so-called natural, bioactive hydrogel-based bio-inks. Mostly, endowing the bio-inks with bioactive properties will facilitate improvements in the constructs’ ability to bind with the host tissue at the implantation site [431].

## Figures and Tables

**Table 2:** Comparison of different bioprinting techniques.

Parameters	Bioprinting Approaches			
	Extrusion-Based	Inkjet-Based	Laser-Assisted	Vat Polymerization-Based
Printing process	Line-by-line	Drop-by-drop	Dot-by-dot	Layer-by-layer
Bio-ink viscosity	30–6 × 10^7^ mPa·s	3.5–12 mPa·s	1–300 mPa·s	No limitation
Resolution	200–1000 μm	10–50 μm	10–100 μm	<50 μm
Post-printing cell viability	40–80%	>85%	>95%	>85%
Cell density	High Cell spheroids	Low <10^6^ cells·mL^−1^	Medium ~10^8^ cells·mL^−1^	Medium ~10^8^ cells·mL^−1^
Printing speed	Slow 10–700 mm·s^−1^	Fast >10^3^ droplets·s^−1^	Moderate 200–1600 mm·s^−1^	Fast Multi layers·s^−1^

**Table 3:** Major parameters that influence the performances of different bioprinting techniques.

Printing Technique	Parameters That Influence Printing Performance	References
Inkjet-based 3D printing	The performance of this printing technique may be influenced by parameters such as printing speed and ink formulation. Indeed, the printing speed can be a challenge for constructing millimeter or centimeter scale biostructures, as maintaining cell viability during many hours of printing is very demanding. Bio-ink formulation influences its volumetric flow rate (i.e., the bio-ink volume that passes through the needle or nozzle per unit of time) and is essential to determine the shape of bioprinted filaments or droplets. In general, higher flow rates are associated with lower printing speeds, leading to an increase in the filament diameter. On the contrary, small flow rates combined with higher printing speeds reduce the filament diameter. Another factor that influences inkjet-based 3D printing is nozzle/extrusion temperature. This is because this parameter dictates the layer thickness of the ink, the printing fidelity and the durability of the cells.	[186,187,188,189]
Extrusion-based 3D printing	Factors such as the applied pressure, nozzle orifice size and geometry play a critical role regarding the printing outcome, since these factors can influence the construct properties such as layer thickness and building orientation. They are also dominant factors that may cause cell damage when printing cell-laden hydrogels. It has been confirmed that cell mortality upon printing is proportional to the nozzle diameter and system pressure employed (increased printing pressure decreases cell viability).	[160,190,191,192,193,194]
Laser-assisted 3D printing	This printing technique is influenced by parameters such as ink formulation, extruder temperature and laser orientation. Ink formulation is particularly relevant since, in addition to influencing the flowrate of the bio-ink, it also influences the rheological properties of bio-inks. These properties dictate printing fidelity, flow behavior, viscosity, shear stress and viscoelasticity of the bio-ink and viability of the cells.	[70,192,193,195,196,197]
Vat polymerization-based printing	The performance of this printing technique will be influenced by parameters such as rheological properties, layer thickness, post-curing time and orientation. Other factors such as exposure time to determine the exposure duration of a single layer, lifting height and speed and lowering speed are crucial parameters that influence the photopolymerization of the associated biopolymer and thus are crucial to determining the printing fidelity.	[198,199,200]

**Table 4:** Application of natural hydrogel-based bio-inks in the regeneration of several types of damaged tissues.

Tissues or Organs	Bio-Inks	References
Cartilage tissue	Cartilage-derived dECM, mixed with chondrocytes and converted into a photo-crosslinkable hydrogel using methacrylation.	[78]
	Agarose hydrogel was seeded with mesenchymal stem cells.	[67]
	Human nasal chondrocytes with agarose hydrogel.	[69]
	Chondrocytes seeded in nanocellulose–alginate hydrogel.	[63]
	Alginate-based hydrogel embedded with human mesenchymal stem cells.	[142]
	Hyaluronic acid and alginate hydrogel with human articular chondrocytes.	[50]
	Cartilage-resident gelatin methacryloyl hydrogel was laden with chondroprogenitor cells.	[136]
	Nanocellulose hydrogel laden with human chondrocytes.	[66]
	Fibroblasts with nanocellulose-alginate hydrogel	[49]
	Chondrocytes with gelatin-hyaluronic acid hydrogel, bioprinted and crosslinked during the deposition process to obtained sculpted 3D structures.	[275]
	Carrageenan hydrogel laden with chondrogenic cells.	[72]
	Silk-based hydrogel loaded with platelet-rich plasma (PRP).	[300]
	Gelatin methacryloyl-based hydrogels with chondroprogenitor cells, mesenchymal stromal cells and chondrocytes.	[209]
Skin tissue	Fibroblasts with nanocellulose-alginate based hydrogel.	[49]
	dECM-based hydrogel with multiple cell types.	[30]
	Hydrogel based on dECM laden with endothelial progenitor cells and adipose-derived stem cells.	[90]
	Collagen hydrogel with enveloped keratinocytes and fibroblasts.	[91]
	Gelatin-methacryloyl hydrogel laden with human fibroblasts.	[92]
Neural tissue	Human-induced pluripotent stem cells encapsulated within the fibrin-based hydrogel.	[129]
	Schwann cells embedded in methacrylated hyaluronic acid and collagen hydrogel.	[57]
	Fibrin-based hydrogel incorporated with neural progenitor cells.	[126]
	Neural cells embedded within a fibrin-based hydrogel aimed at the modeling of brain tissue.	[127]
Chondral tissue	Human mesenchymal stromal cells incorporated into collagen and supramolecular hyaluronic acid hydrogel matrix.	[54]
	Stem cells embedded within silk-based hydrogel.	[133]
	Surgical printing at a chondral wound site of human adipose stem cells seeded in gelatin–methacrylamide hydrogel combined with methacrylated hyaluronic acid hydrogel.	[301]
	Alginate hydrogel with incorporated human chondrocytes and osteogenic progenitors.	[302]
Blood vessels	Encapsulation of fibroblasts in sausage-like crosslinked hydrogel comprising polyethylene glycol, hyaluronic acid and gelatin.	[53]
	Vascular smooth muscle cell–laden hydrogel comprising gelatin methacryloyl, polyethylene(glycol) diacrylate and alginate.	[93]
	Multiple cell types embedded in gelatin methacryloyl hydrogel.	[94]
Muscle tissue	Human skeletal muscle cells seeded in dECM-based hydrogel.	[81]
	Progenitor cells seeded in dECM-based hydrogel.	[121]
	Primary human airway and intestinal smooth muscle cells seeded in alginate-based matrix with either collagen or intestinal dECM.	[125]
Bone tissue	Alginate-gelatin-agarose hydrogel laden with SaOS-2 cells.	[32]
	Human osteosarcoma cells seeded in bone-like hybrid hydrogel comprising chitosan and hydroxyapatite nanocrystals.	[85]
	Osteoblast cells incorporated in chitosan hydrogel.	[86]
	Silk-gelatin hydrogel embedded with mesenchymal stem cells.	[297]
Biological engineered tissues	Induced pluripotent stem cells contained in crosslinked hydrogel comprising alginate, chitosan and agarose.	[46]
	Platelet-rich plasma encapsulated in alginate-gelatin hydrogel.	[101]
	Agarose hydrogel mixed with human mesenchymal stem cells.	[70]
	Hyaluronic acid a collagen derivative hydrogel containing human bone marrow–derived mesenchymal stromal cells.	[102]
Cardiac tissue	Human-induced pluripotent and mesenchymal stem cells loaded with dECM.	[84]
	Alginate hydrogel containing human cardiac-derived cardiomyocyte progenitor cells.	[131]
Periodontal tissue	Gelatin-alginate hydrogel with human dental pulp stem cells.	[122]
	Human primary periodontal ligaments cells with gelatin-methacryloyl hydrogel.	[130]
Renal tissue	Human kidney cells with photo-crosslinkable dECM, chemically modified by methacrylation.	[29]
	Alginate, gelatin and pectin hydrogel loaded with epithelial endothelial cells.	[128]
Adipose tissue	Human adipose-derived mesenchymal incorporated into a gelatin-alginate hydrogel.	[96]
	Human adipose-derived stem cell–laden dECM hydrogel.	[83]
Tracheal graft	Mesenchymal stem cells seeded in fibrin hydrogel, with coated 3D bioprinting polycaprolactone scaffold.	[37]
Vaginal wall	Endometrial mesenchymal stem cells embedded in the matrix, alginate-based hydrogel.	[132]
Breast tissue	Human adipose-derived stem cells with dECM hydrogel.	[99]
Vascular constructs	Fibrinogen-gelatin hydrogel with primary neonatal human dermal fibroblasts.	[303]
Menisci	Silk–gelatin hydrogel seeded with fibrochondrocytes.	[38]
Spinal cord	Collagen-silk hydrogel with incorporated neural stem cells.	[298]

## Data Availability

Not appicable.
